# Lead‐Free Halide Double Perovskite Nanocrystals for Light‐Emitting Applications: Strategies for Boosting Efficiency and Stability

**DOI:** 10.1002/advs.202004118

**Published:** 2021-03-03

**Authors:** Huidong Tang, Yanqiao Xu, Xiaobo Hu, Qing Hu, Ting Chen, Weihui Jiang, Lianjun Wang, Wan Jiang

**Affiliations:** ^1^ School of Material Science and Engineering Jingdezhen Ceramic Institute Jingdezhen 333001 P. R. China; ^2^ Engineering Research Center of Advanced Glasses Manufacturing Technology Ministry of Education Donghua University Shanghai 201620 P. R. China; ^3^ National Engineering Research Center for Domestic and Building Ceramics Jingdezhen 333001 P. R. China

**Keywords:** efficiency and stability, lead‐free halide double perovskite nanocrystals, light‐emitting diodes, self‐trapped exciton

## Abstract

Lead‐free halide double perovskite (HDP) nanocrystals are considered as one of the most promising alternatives to the lead halide perovskite nanocrystals due to their unique characteristics of nontoxicity, robust intrinsic thermodynamic stability, rich and tunable optoelectronic properties. Although lead‐free HDP variants with highly efficient emission are synthesized and characterized, the photoluminescent (PL) properties of colloidal HDP nanocrystals still have enormous challenges for application in light‐emitting diode (LED) devices due to their intrinsic and surface defects, indirect band, and disallowable optical transitions. Herein, recent progress on the synthetic strategies, ligands passivation, and metal doping/alloying for boosting efficiency and stability of HDP nanocrystals is comprehensive summarized. It begins by introducing the crystalline structure, electronic structure, and PL mechanism of lead‐free HDPs. Next, the limiting factors on PL properties and origins of instability are analyzed, followed by highlighting the effects of synthesis strategies, ligands passivation, and metal doping/alloying on the PL properties and stability of the HDPs. Then, their preliminary applications for LED devices are emphasized. Finally, the challenges and prospects concerning the development of highly efficient and stable HDP nanocrystals‐based LED devices in the future are proposed.

## Graphical abstract

A review of lead‐free halide double perovskite (HDP) nanocrystals is presented to introduce the crystal and electronic structure, and highlight the recent progress on the synthetic strategies, ligands passivation, and metal doping/alloying for boosting their efficiency and stability. The outlook on developing high‐performance lead‐free HDP nanocrystals toward light emitting diodes are conceived and expected to be of great significance for guiding future research.

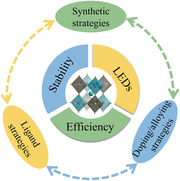

## 1 Introduction

In recent years, lead halide perovskite nanocrystals, APbX_3_ (A = CH_3_NH_3_^+^(MA^+^), NH_2_HCNH_2_^+^ (FA^+^), and Cs^+^, X = Cl, Br, and I) have attracted great interest and been widely used in solar cells,^[^ ^1^, ^2^ ^]^ lasers,^[^ ^3^, ^4^ ^]^ light‐emitting diodes (LEDs),^[^ ^5^, ^6^ ^]^ bioimaging^[^ ^7^ ^]^ and photodetectors^[^ ^8^, ^9^ ^]^ due to their broad excitation, narrow‐band emission (full width at half‐maximum (FWHM) of 12–42 nm),^[^ ^10^, ^11^ ^]^ tunable wavelength (400–700 nm),^[^ ^12^, ^13^, ^14^ ^]^ high photoluminescence quantum yield (*PLQY* ≈ 100%),^[^ ^15^, ^16^ ^]^ direct bandgap,^[^ ^17^ ^]^ defect tolerance,^[^ ^18^ ^]^ long diffusion length,^[^ ^19^, ^20^ ^]^ high carrier mobility,^[^ ^21^ ^]^ and long carrier lifetime.^[^ ^22^, ^23^ ^]^ Especially, lead halide perovskite nanocrystals are suitable for solid‐state lighting and high‐definition display applications due to their wide color gamut (*NTSC* ≈ 140%),^[^ ^24^, ^25^ ^]^ high color purity,^[^ ^26^ ^]^ as well as facile synthesis methods.^[^ ^27^, ^28^, ^29^, ^30^ ^]^ In 2014, Tan and co‐workers first reported MAPbBr_3_ and MAPbI_3−_ *_x_*Cl*_x_* as emitters in LED devices with external quantum efficiency (*EQE*) of 0.1% and 0.76%, respectively.^[^ ^31^ ^]^ In the following 6 years, the performance of LEDs has been remarkably enhanced, among which *EQE* increased from 0.1% to 16.3%.^[^ ^32^, ^33^, ^34^ ^]^ Most recently, several groups have reported highly efficient LEDs with *EQE* over 20%.^[^ ^35^, ^36^, ^37^, ^38^ ^]^ For instance, Xiao group^[^ ^37^ ^]^ reported that high‐property LEDs with a *EQE* of 20.9% and 694 nm emission wavelength could be obtained using FA_0.33_Cs_0.67_Pb(I_0.7_Br_0.3_)_3_ as emitters. Song group^[^ ^38^ ^]^ realized a 21.63% *EQE* record of green emission perovskite LEDs by designing bilateral electron transport structure. Despite the fast development of APbX_3_ nanocrystals, poor stability under moisture, oxygen, heat, and light conditions, as well as intrinsic toxicity of lead ion have been becoming mainly obstacle for their commercial application in the LED devices.^[^ ^39^, ^40^ ^]^ Hence, the designing of lead‐free halide perovskite nanocrystals with excellent photoelectric properties and higher stability has important theoretical and practical significance. So far, various lead‐free halide perovskite nanocrystals have been synthesized and characterized, such as CsB^2+^X_3_ (B = Sn^2+^, Ge^2+^, Yb^2+^, and Eu^2+^) nanocrystals,^[^ ^41^, ^42^, ^43^, ^44^ ^]^ Cs_3_B^3+^_2_X_9_ (B = Sb^3+^, Bi^3+^) nanocrystals,^[^ ^45^, ^46^ ^]^ and Cs_3_Cu_2_X_5_ (X = Cl, Br, and I) nanocrystals.^[^ ^47^, ^48^ ^]^ However, these emerging nanocrystals are still suffering from the problems of instability, larger bandgap, and defect intolerance.

Generally, lead‐free halide double perovskite (HDP, A_2_B^+^B^3+^X_6_) nanocrystals consisting of one monovalent (B^+^) and one trivalent (B^3+^) cation have more degrees of freedom in selecting the B^+^/B^3+^‐cation elements, high electronic dimensionality, and nontoxicity,^[^ ^49^, ^50^ ^]^ which are desirable alternatives for lead‐based perovskite nanocrystals. Since 2018, Creutz et al.^[^ ^51^ ^]^ and Han group^[^ ^52^ ^]^ successfully synthesized Cs_2_AgBiX_6_ (X = Cl, Br, and I) nanocrystals via modified high temperature hot injection method and room temperature anion exchange method as well as antisolvent recrystallization method, which exhibited adjustable emission wavelength in the range of 395–575 nm. The colloidal HDP nanocrystals have made great progress in preparation technology and performance modulation, showing rich and tunable optoelectronic properties, robust stability, low exciton binding energies, and high absorption efficiency. Lead‐free HDP nanocrystals have been documented wide application prospects in solar cells,^[^ ^53^, ^54^, ^55^, ^56^, ^57^ ^]^ LEDs,^[^ ^58^ ^]^ photocatalysis,^[^ ^59^, ^60^ ^]^ photodetectors,^[^ ^61^ ^]^ and many other optoelectronic fields.^[^ ^62^, ^63^ ^]^ Although Sn^4+^‐based,^[^ ^64^, ^65^, ^66^, ^67^ ^]^ Zr^4+^‐based,^[^ ^68^ ^]^ Na^+^/Bi^3+^‐based,^[^ ^69^, ^70^ ^]^ Na^+^/In^3+^‐based,^[^ ^71^ ^]^ K^+^/In^3+^‐based,^[^ ^71^ ^]^ Ag^+^/In^3+^‐based,^[^ ^72^, ^73^, ^74^, ^75^, ^76^, ^77^ ^]^ and Mn^2+^/Bi^3+^‐based,^[^ ^78^, ^79^ ^]^ lead‐free HDP bulk materials with highly efficient emission (**Table** **1**) have been designed and synthesized in recent years, the photoluminescent (PL) properties of colloidal HDP nanocrystals still remain enormous challenge for application in LED devices compared with lead halide perovskite nanocrystals. The main reasons for poor PL performance of colloidal HDP nanocrystals can be ascribed to their intrinsic and surface defects, indirect band gaps, and forbidden transitions. To fundamentally settle the above issues, synthesis strategies, ligands passivation, and metal doping/alloying have been put forward for boosting or modulating photoelectric performance of lead‐free HDP nanocrystals. At present, several review articles about double perovskite materials have been published. It is remarkable that these reviews are mainly focused on the synthesis, optical performance, and application of lead‐free halide perovskite materials.^[^ ^80^, ^81^, ^82^, ^83^, ^84^ ^]^ But these reviews rarely introduce HDP nanocrystals. The existing several reviews only provide a brief introduction on synthesis, stability, band gap engineering, doping, and application of currently studied HDP nanocrystals.^[^ ^85^, ^86^, ^87^, ^88^ ^]^ However, the strategies for improving optical properties and stability of colloidal HDP nanocrystals have not been summarized systematically.

**Table 1:** PL properties and synthesis method of lead‐free HDP variants

Chemical components	Emission peak [nm]	*PLQY* [%]	Synthesis method	Ref.
Cs_2_SnCl_6_:Ce^3+^	455	6.54	Precipitation method	^[^ ^64^ ^]^
Cs_2_SnCl_6_:Bi^3+^	455	78.9	Hydrothermal method	^[^ ^65^ ^]^
Cs_2_SnCl_6_:Sb^3+^	602	37	Hydrothermal method	^[^ ^66^ ^]^
Cs_2_SnCl_6_:Te^4+^	580	95.4	Hydrothermal method	^[^ ^67^ ^]^
Cs_2_ZrCl_6_:Bi^3+^	456	50	Hydrothermal method	^[^ ^68^ ^]^
Cs_2_NaBiCl_6_:Mn^2+^	590	15	Precipitation method	^[^ ^69^ ^]^
Cs_2_NaBi_1−_ *_x_*In*_x_*Cl_6_:0.03Mn^2+^ (x = 0–1.0)	577–585	56	Hydrothermal method	^[^ ^70^ ^]^
Cs_2_NaInCl_6_:Sb^3+^	445	82	Precipitation method	^[^ ^71^ ^]^
Cs_2_KInCl_6_:Sb^3+^	495	93	Precipitation method	^[^ ^71^ ^]^
Cs_2_AgInCl_6_:Bi^3+^	600 ± 30	34 ± 4	Precipitation method	^[^ ^72^ ^]^
Cs_2_AgInCl_6_:Cr^3+^	1010	23.5	High temperature solid‐state reaction	^[^ ^73^ ^]^
Cs_2_Ag_0.6_Na_0.4_InCl_6_:Bi^3+^	565	86 ± 5	Hydrothermal method	^[^ ^74^ ^]^
Cs_2_Ag_0.6_Na_0.4_InCl_6_:Ho^3+^	490, 550, and 650	60.49	Hydrothermal method	^[^ ^75^ ^]^
Cs_2_Ag_0.4_Na_0.6_InCl_6_:Bi^3+^‐Ce^3+^	570	98.6	Precipitation method	^[^ ^76^ ^]^
Cs_2_Na_0.4_Ag_0.6_ In_0.995_Bi_0.005_Cl_6_:Mn^2+^	550, 610	31.8	Hydrothermal method	^[^ ^77^ ^]^
Cs_4_MnBi_2_Cl_12_	610	25.7	Hydrothermal method	^[^ ^78^ ^]^
Cs_4_CdBi_2_Cl_12_:Mn^2+^	605	57	Precipitation method	^[^ ^79^ ^]^

Herein, this paper summarizes the strategies to boost the emission efficiency and stability of lead‐free HDP nanocrystals, which are expected to be significant guiding for designing and fabricating highly efficiency, stability, and nontoxicity LED devices. The crystalline structure, electronic structure, and PL mechanism of lead‐free HDP nanocrystals are first introduced. Then, we analyze the limiting factors on optical properties and sources of instability. At the same time, we focus on the effects of synthesis strategies, ligands passivation, and metal cation doping/alloying on the photoelectric performance and stability of the HDP nanocrystals. In addition, lead‐free HDP materials for LED applications are emphasized. In the last part, we outline the challenges and prospects concerning the development of highly efficient and stable HDP nanocrystals‐based LED devices in the future.

## 2 Crystal and Electronic Structure of Halide Double Perovskites

### 2.1 Crystal Structure

**Figure 1:**
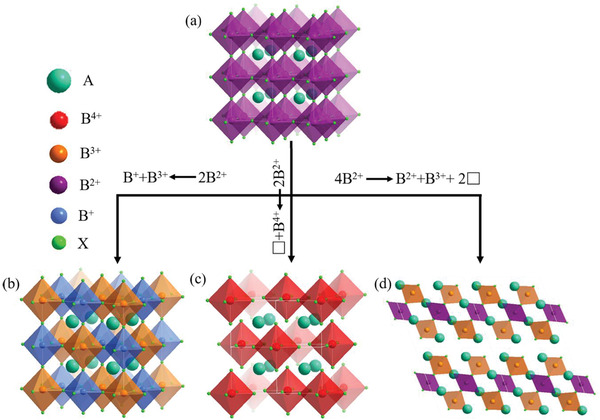
The crystal structures of a) simple halide perovskites (AB^2+^X_3_), b) halide double perovskites (A_2_B^+^B^3+^X_6_), c) vacancy‐ordered halide double perovskites (A_2_B^4+^X_6_), and d) layered halide double perovskites (A_4_B^2+^B^3+^_2_X_12_).

To overcome the toxicity of Pb^2+^ ions, the cation‐transmutation is a very effective and straightforward strategy. The 3D HDPs with a general chemical formula A_2_B^+^B^3+^X_6_ (i.e., K_2_NaAlF_6_ elpasolite structure) can be obtained by converting two divalent cations (Pb^2+^) into a B^+^and B^3+^ in halide perovskite lattice.^[^ ^89^ ^]^ Theoretically, A^+^ site may be monovalent cation such as Cs^+^ and Rb^+^, B^+^ may be monovalent cation such as K^+^, Na^+^, and Ag^+^, B^3+^ may be trivalent cation such as Bi^3+^, In^3+^, and Sb^3+^, and X^−^ may be halide ion such as I^−^, Br^−^, and Cl^−^.^[^ ^90^, ^91^, ^92^ ^]^ **Figure** **1**a,b display the crystal structure of AB^2+^X_3_ and A_2_B^+^B^3+^X_6_ 3D halide perovskites, respectively. The B^+^ and B^3+^ cations with large charge difference exhibit alternating arrangement in the octahedral cavity and make up sixfold coordination with the halide ions, respectively. The A site cations are located in cavities formed by octahedrons. In addition, the two divalent cations Pb^2+^ are substituted via a vacancy and a quadrivalent cation such as Sn^4+^, Ge^4+^,and Ti^4+^, resulting in the formation of A_2_B^4+^X_6_ (i.e., K_2_PtCl_6_ prototype structure), which can be named as “vacancy‐ordered double perovskites”.^[^ ^93^, ^94^ ^]^ The crystal unit of vacancy‐ordered double perovskites is very similar to that of the double perovskites. Specially, the vacancy‐ordered double perovskites have 50% periodic vacancies on the octahedral cavity, as shown in Figure 1c. If the four Pb^2+^ ions are substituted by two vacancies, a divalent cation and two trivalent cations, the layered HDPs with defective 3D structure can be formed.^[^ ^95^, ^96^ ^]^ It is the general chemical formula of A_4_B^2+^B^3+^_2_X_12_, where B^2+^ could be Sn^2+^, Ge^2+^ and Cu^2+^, B^3+^ could be Sb^3+^, In^3+^ and Bi^3+^.^[^ ^97^ ^]^ The layered HDPs are a unique hybrid metal <111>‐oriented layered perovskites, which contain a B^2+^X_6_ octahedra inserted between two layers of B^3+^X_6_ octahedrons. In other words, the vacancies generated by B^2+^ site cations, resulting in a collapse of the 3D‐network into a 2D network (see Figure 1d).^[^ ^98^ ^]^ In summary, the HDPs from hetero‐substitution of Pb^2+^ ions can maintain the high structural dimensionality and offer flexibility for various chemical compositional adjustments, which provide more probability for discovering and designing new perovskite structure.

### 2.2 Electronic Structure

**Figure 2:**
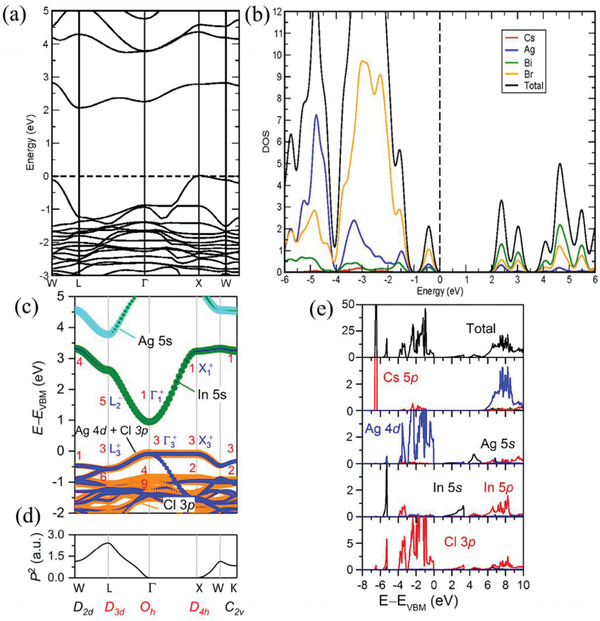
a) PBE calculation band structures and b) projected densities of states for Cs_2_AgBiBr_6_ double perovskites_._ Reproduced with permission.^[^ ^106^ ^]^ Copyright 2016, American Chemical Society. c) Band structures, d) transition matrix elements, and e) projected densities of states of Cs_2_AgInCl_6_ double perovskites by PBE calculation_._ Reproduced with permission.^[^ ^109^ ^]^ Copyright 2017, American Chemical Society.

The optoelectronic properties of HDPs strongly depend on the bandgap and electronic structures, which are related to the atomic orbitals and occupation sites of the B^+^ and B^3+^ cations.^[^ ^99^, ^100^ ^]^ It has been found that the lead halide perovskite nanocrystals with eminent photoelectric performance are based on the 6s^2^ lone‐pair states of Pb^2+^ ions.^[^ ^101^, ^102^ ^]^ Therefore, whether both the B^+^ and B^3+^ cations possess lone‐pair states are important for improving optoelectronic properties of HDP nanocrystals.^[^ ^99^, ^101^ ^]^ Several ideal candidates with both lone‐pair states and suitable direct bandgap have been investigated in theory, such as Cs_2_InSbCl_6_ (0.98 eV),^[^ ^103^, ^104^ ^]^ Cs_2_InBiCl_6_ (0.88 eV),^[^ ^103^, ^104^ ^]^ and Cs_2_TlInI_6_ (1.37 eV).^[^ ^105^ ^]^ Unfortunately, the In^1+^ based HDPs are extremely instability due to the easy oxidization of In^1+^ to In^3+^. The other concern is Tl^1+^ based HDPs, which are even more toxic than lead halide perovskites.^[^ ^105^ ^]^ In addition, only one of the B^+^ or B^3+^ cations for some HDPs (e.g., Cs_2_AgBiX_6_) has lone‐pair states. For instance, the Cs_2_AgBiCl_6_ and Cs_2_AgBiBr_6_ possess band gaps in the range from 2.22 to 2.77eV and 1.9 to 2.19 eV,^[^ ^106^, ^107^ ^]^ respectively. The conduction band minimum (CBM) of Cs_2_AgBiBr_6_ mainly consists of Ag 5s, Bi 6p, and Br 4p orbitals at the L point and the valence band maximum (VBM) mainly derives from Ag 4d, Bi 6s, and Cl 4p states at the X point (**Figure** **2**a,b).^[^ ^106^ ^]^ Thus, the Cs_2_AgBiBr_6_ exhibits indirect bandgap, leading to a large carrier effective masse and a low the radiative recombination. On the other hand, both B^+^ and B^3+^ cations in some HDPs such as Cs_2_AgInCl_6_, do not contain lone‐pair s^2^ states. The Cs_2_AgInCl_6_ exhibits a direct band gap of 1.03 eV at the Γ point by Perdew–Bruke–Ernzerhof (PBE) calculation results (Figure 2c,d).^[^ ^108^ ^]^ The Heyd‐Scuseria‐Ernzerhof hybrid functional (HSE06) can calibrate the obvious underestimation of the PBE band gap, which outputs 3.51 eV for Cs_2_InAgCl_6_.^[^ ^72^ ^]^ The CBM is mainly comprised of the delocalized In 5s states, while the VBM consists of Ag 4d and Cl 3p orbitals (Figure 2e).^[^ ^109^ ^]^ However, the electronic transition from VBM to CBM in Cs_2_AgInCl_6_ is parity forbidden due to angular momentum conservation, which gives birth to a larger optical band gap.^[^ ^109^ ^]^ These results indicated that HDP nanocrystals might be difficult to be directly applied in optoelectronic field.

**Figure 3:**
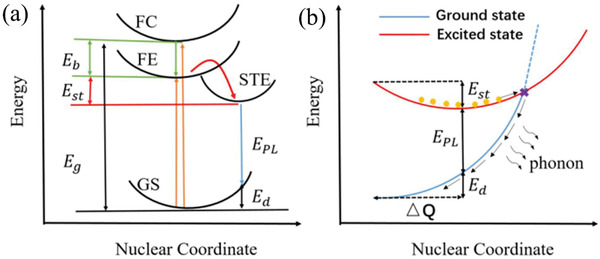
a) The schematic diagram of STEs (GS, ground state; FE, free exciton state; FC, free carrier state;) and b) schematic of the nonradiative recombination process for STE when *S* is large. Orange circles represent excited electrons. Reproduced with permission.^[^ ^112^ ^]^ Copyright 2019, American Chemical Society.

The PL mechanism of most HDP materials, such as Cs_2_SnCl_6_,^[^ ^65^ ^]^ Cs_2_AgInCl_6_,^[^ ^74^ ^]^ and Cs_2_AgBiBr_6_^[^ ^110^ ^]^ belongs to exciton luminescence, which can be described as electrons and holes attracting each other and recombining to emit photon under Coulomb interaction. The self‐trapped excitons (STEs) usually occur in soft lattice and strong electron‐phonon coupling of halide perovskite materials, and their emission energy is seen to be significantly smaller than the bandgap. The formation of STEs can be evaluated by Huang–Rhys factor *S*, which can be expressed as Equation (1):^[^ ^111^ ^]^

(1) $$\mathrm{FWHM}=2.36\sqrt{S}\hbar \omega_{\mathrm{phonon}}\sqrt{\coth\frac{\hbar \omega_{\mathrm{phonon}}}{2K_{B}T}}$$

where ℏ is Planck constant, *ω*_phonon_ is phonon frequency, *K*_B_ is Boltzmann constant, and *T* is temperature. It is certain that the *S* value is positively correlated with FWHM and the larger *S* value is easier to form STEs. Hence, the PL emission is broadband spectrum and allows a large Stokes shift. The emission energy (*E*_PL_) can be calculated to by *E*_PL_ = *E*_g_ − *E*_b_ − *E*_st_ − *E*_d_, where *E*_g_ is bandgap energy, *E*_b_ is exciton binding energy, and *E*_st_ is self‐trapping energy, as well as *E*_d_ is lattice deformation energy (**Figure** **3**a).^[^ ^112^ ^]^ The coordinate difference (Δ*Q*) between the free‐exciton and STEs, which is directly proportional to *S*. However, larger *S* value indicates the easier transformation from excited state and ground state to the cross (Figure 3b), which means that some phonons might loss via nonradiative recombination from some exciton electrons and holes.^[^ ^112^ ^]^ Therefore, the suitable value of *S* is conducive to obtaining efficient STE emission. For instance, Cs_2_AgInCl_6_ possesses white emission at 590 nm from STEs, and the emission wavelengths can cover 400 to 800 nm.^[^ ^65^ ^]^ The study found that the wave function of electrons with a 3D structure was dispersive due to the delocalized In 5s states, while the holes with a 0D structure were strongly confined at [AgCl_6_] octahedral. After excitation, the holes would be quickly trapped at [AgCl_6_] octahedra and the electronic configuration of Ag would be changed from 4d^10^ to 4d^9^, resulting in a strong Jahn–Teller distortion of the AgCl_6_ octahedron and further formation of the STEs. However, the *PLQY* of Cs_2_AgInCl_6_ was relatively poor (<0.1%) because STEs were parity‐forbidden transitions. Luo et al.^[^ ^65^ ^]^ proposed that partially substitution of substituted for Ag^+^ with Na^+^ in Cs_2_AgInCl_6_ could break the dark transition by changing the parity of the STEs wavefunction, thereby *PLQY* was significantly improved. In addition, Cong et al.^[^ ^113^ ^]^ thought that the Cs_2_AgBiCl_6_ nanocrystals with indirect bandgap possessed strong exciton‐phonon coupling, which caused non‐radiative STEs, while Cs_2_AgBi_0.1_In_0.9_Cl_6_ nanocrystals with direct bandgap had moderate exciton‐phonon coupling, which produced bright STEs emission. At present, the application of STEs emission in HDP is also very common phenomenon, which is beneficial to understand PL mechanism of HDP nanocrystals thoroughly.

In summary, the HDP materials currently face two issues by analyzing electronic and band structure: i) The HDP nanocrystals with excellent optoelectronic properties are difficult to achieve by existing synthesis strategies due to their poor stability. ii) Some HDP materials with excellent stability have poor optoelectronic properties due to indirect band gaps and parity‐forbidden transitions. Thus, many strategies have been proposed to realize or boost the stability, nontoxicity, and excellent optoelectronic properties of HDP nanocrystals.

## 3 Strategies for Boosting the Efficiency of Halide Double Perovskite Nanocrystals

### 3.1 Synthetic Strategies

To prepare and explore high quality lead‐free HDP nanocrystals with a controllable composition, shape, size, and properties, tremendous efforts have been devoted to develop convenient, low‐cost, and reliable synthetic strategies. These methods mainly include high temperature hot‐injection technique, room temperature anti‐solvent recrystallization method, and halide ion exchange reactions, as summarized in **Table** **2**. In this section, we will focus on the synthesis pathway and their influence on the shape and size of lead‐free HDP nanocrystals.

**Table 2:** Summary of the morphology, synthetic strategies, and ligands of HDP nanocrystals

Nanocrystals	Morphology	Synthetic strategies	Ligands	Ref.
Cs_2_AgBiCl_6_	Cube/quasi‐spherical shapes	Modified hot‐injection approach (by injecting TMSCl)/anti‐solvent recrystallization (isopropanol as anti‐solvent)/variable temperature hot injection method	OA, OLA/OA, OLA/OA	^[^ ^51^, ^52^, ^130^ ^]^
Cs_2_AgBiBr_6_	Cube/quasi‐spherical shapes	Hot‐injection (by injecting Cs‐Oleate)/modified hot‐injection approach (by injecting TMSBr)/anti‐solvent recrystallization method (isopropanol as anti‐solvent)	OA, OLA/OA, OLA/OA	^[^ ^51^, ^52^, ^120^ ^]^
Cs_2_AgBiI_6_	Cube/quasi‐spherical shapes	Anion exchange/anti‐solvent recrystallization method (isopropanol as anti‐solvent)	OA, OLA/OA	^[^ ^51^, ^52^ ^]^
Cs_2_AgInCl_6_	Cube shapes	Modified hot‐injection approach (by injecting BzCl)	OA, OLA	^[^ ^127^, ^128^ ^]^
Cs_2_NaInCl_6_	Cube shapes	Variable temperature hot injection method (by injecting TMSCl)	OA, OLA	^[^ ^130^ ^]^
Cs_2_AgSbCl_6_	Cube/spherical shapes	Modified hot‐injection approach (by injecting BzCl)/variable temperature hot injection (by injecting TMSCl)/hot‐injection method (by injecting Cs‐Oleate)/ligand‐assisted reprecipitation technique (ethyl acetate as anti‐solvent)	OA, OLA/OA, OLA/OA	^[^ ^121^, ^128^, ^130^, ^137^ ^]^
Cs_2_AgSbBr_6_	Cube shapes	Ligand‐assisted reprecipitation technique (ethyl acetate as anti‐solvent)/modified hot‐injection method (by injecting Cs‐Oleate))	OA/OA, OLA	^[^ ^121^, ^137^ ^]^
Cs_2_AgSbI_6_	‐	Ligand‐assisted reprecipitation technique (ethyl acetate as anti‐solvent)	OA	^[^ ^137^ ^]^
Cs_2_NaBiCl_6_	Quasi‐spherical shapes/cuboctahedral/cuboidal Cuboctahedral shapes	Modified hot injection approach (by injecting TMSCl)	OA, OLA	^[^ ^124^, ^125^, ^126^ ^]^
Cs_2_NaBiBr_6_	Cuboctahedral/cuboidal	Modified hot injection approach (by injecting TMSBr)	OA, OLA	^[^ ^125^, ^126^ ^]^
Cs_2_SnCl_6_	Quasi‐spherical shapes	Hot‐injection method (by injecting Cs‐Oleate)	OA, OLA	^[^ ^119^ ^]^
Cs_2_SnI_6_	Dot/nanorods/nanowires/nanobelts/nanoplatelets shapes	Hot‐injection method (by injecting Cs‐Oleate) /ultrasonic irradiation aqua low temperature method	OA, OLA/Triphenylphosphite	^[^ ^117^, ^118^, ^138^ ^]^
Cs_4_CuSb_2_Cl_12_	Spherical like/dot shapes	Modified hot injection approach (by injecting TMSCl)/ultrasonic exfoliation method	OA, OLA	^[^ ^129^, ^139^ ^]^

#### 3.1.1 Hot Injection Technique

The hot injection method is based on the quickly injection of a precursor into another mixture solution consisting of another precursors, ligands, and high‐boiling point non‐polar solvent, which is performed at an elevated temperature and protective gas.^[^ ^30^, ^114^ ^]^ The reaction only needs a few seconds due to fast nucleation and growth kinetics, resulting in the formation of nanocrystals with high crystallinity and good monodispersity.^[^ ^115^ ^]^ In addition, the attaining isolation between the nucleation and growth stages endows the synthesized lead‐free HDP nanocrystals with a narrow size distribution.^[^ ^116^ ^]^ In 2015, Protesescu and co‐workers first introduced the high temperature hot injection method to successfully synthesize CsPbX_3_ perovskite nanocrystals.^[^ ^24^ ^]^ This method can also be employed to prepare lead‐free HDP nanocrystals with some modification due to the similar features of the perovskite family. In 2016, Wang et al.^[^ ^117^ ^]^ first reported the synthesis of colloidal Cs_2_SnI_6_ vacancy‐ordered HDP nanocrystals via hot injection method using oleic acid (OA) and oleylamine (OLA) as primary ligands as well as octadecene (ODE) as a high‐boiling point non‐coordinating solvent at inert atmosphere. Typically, the Cs‐oleate was prepared by mixing Cs_2_CO_3_, OA, and ODE in a protective atmosphere with specific temperature (such as 150 °C under N_2_ atmosphere) and pre‐heated at 120 °C before using, as shown below (Equation (2)):

(2) $$\begin{array}{ccc} & & Cs_{2}CO_{3}+2\mathrm{RCOOH}\;\overset{150\;{}^{\circ }C\;\mathrm{under}}{\underset{N_{2}\;\mathrm{atmosphere}}{\to }}\;2\mathrm{Cs}\left(\mathrm{RCOO}\right)+CO_{2}+H_{2}O\\ & & \;R=CH_{3}{\left(CH_{2}\right)}_{7}\mathrm{HC}=\mathrm{CH}{\left(CH_{2}\right)}_{7} \end{array}$$

**Figure 4:**
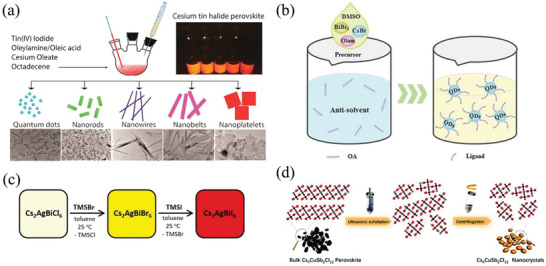
a) Cs_2_SnI_6_ nanocrystals with different shape synthesized by hot‐injection route. Reproduced with permission.^[^ ^117^ ^]^ Copyright 2016, American Chemical Society. b) Reaction scheme of antisolvent recrystallization method. Reproduced with permission.^[^ ^134^ ^]^ Copyright 2017, Wiley‐VCH. c) The synthesis and reaction of Cs_2_AgBiX_6_ nanocrystals by anion exchange. Reproduced with permission. Copyright 2018, American Chemical Society. d) A scheme of the synthesis procedure for Cs_4_CuSb_2_Cl_12_ nanocrystals by ultrasonic exfoliation method. Reproduced with permission.^[^ ^139^ ^]^ Copyright 2019, The Royal Society of Chemistry.

Colloidal Cs_2_SnI_6_ nanocrystals were synthesized after swiftly injecting Cs‐oleate into precursor solution of SnI_4_ salts, which were dissolved in ODE, OLA, and OA (see **Figure** **4**a) at 220 °C. The involved reaction was shown as follows (Equation (3)).^[^ ^118^ ^]^

(3) $$\begin{array}{ccc} 4\mathrm{Cs}\left(\mathrm{RCOO}\right) & + & 2\mathrm{Sn}I_{4\;\;\;}\;\overset{220\;{}^{\circ }C\;\mathrm{under}\;N_{2}\;\mathrm{atmosphere}}{\underset{\mathrm{OA}+\mathrm{OLA}}{\to }}\;Cs_{2}\mathrm{Sn}I_{6}\\ & + & 2\mathrm{CsI}+\mathrm{Sn}{\left(\mathrm{RCOO}\right)}_{4} \end{array}$$

They found that the size and morphology of Cs_2_SnI_6_ nanocrystals were controllable by varying the reaction time, which could realize the selective synthesis of quantum dots, nanorods, nanowires, nanobelts, and nanoplatelets. Specifically, the formation of Cs_2_SnI_6_ nanocrystals was related to reaction temperature. The Cs_2_SnI_6_ nanocrystals with a peak around 620 nm with a FWHM of 49 nm could be synthesized when reaction temperature was beyond 200 °C, while only bulk crystals were obtained at higher reaction temperature (>240 °C).^[^ ^118^ ^]^ Similarly, Cs_2_SnCl_6_ nanocrystals were also prepared by hot injection method with Cs‐oleate injection and SnCl_2_ as starting materials,^[^ ^119^ ^]^ which exhibited blue emission at 438 nm and the *PLQY* was improved to 4.37%.

The hot injection method is further applied for the preparation of lead‐free A_2_B^+^B^3+^X_6_ nanocrystals. In 2018, Zhou et al.^[^ ^120^ ^]^ employed hot injection method to synthesize Cs_2_AgBiBr_6_ nanocrystals by swiftly injecting Cs‐oleate into precursor solution consisting of BiBr_3_, AgNO_3_, ODE, HBr, OA, and OLA at 200 °C (Figure 4b). The involved reaction is shown in the following Equation (4):

(4) $$\begin{array}{ccc} 3\mathrm{BiB}r_{3} & + & 3\mathrm{AgN}O_{3}+4\mathrm{Cs}\left(\mathrm{RCOO}\right)+3\mathrm{HBr}\;\overset{200\;{}^{\circ }C}{\underset{\mathrm{OA}\;+\;\mathrm{OLA}}{\to }}2Cs_{2}\mathrm{AgBiB}r_{6}\\ & + & \mathrm{Ag}\left(\mathrm{RCOO}\right)+\mathrm{Bi}{\left(\mathrm{RCOO}\right)}_{3}+3\mathrm{HN}O_{3} \end{array}$$

The quality of Cs_2_AgBiBr_6_ nanocrystals could be improved by rigorous controlling the amount of HBr, OAand OLA additives as well as the reaction temperature. For example, the small amount HBr could ensure the full ionization of Ag^+^ and effectively hinder the formation of AgBr impurity. The obtained Cs_2_AgBiBr_6_ nanocrystals exhibited well‐defined cubic shape with an average size of 9.5 nm. Similarly, Cs_2_AgSbX_6_ (X = Cl, Br)^[^ ^121^ ^]^ and Cs_2_AgInCl_6_^[^ ^122^ ^]^ nanocrystals with cubic shape were synthesized via hot injection method with Cs‐oleate injection.

In addition, the modified hot‐injection method was proposed for preparing HDP nanocrystals, in which the metal acetates and halide precursors (such as hydrochloric acid, trimethylsilyl chloride (TMSCl), and benzoyl chloride (BzCl)) were used as reaction precursors.^[^ ^123^ ^]^ In other words, the halide precursor was injected into a hot solution of metal acetates. In 2018, Creutz et al.^[^ ^51^ ^]^ first used modified hot‐injection method via injecting TMSX (X = Cl, Br) to synthesize Cs_2_AgBiX_6_ (X = Cl, Br) nanocrystals with a monodisperse cubic shape. It was noteworthy that the nucleation and growth of nanocrystals by injecting trimethylsilyl bromide(TMSBr) were obviously faster than that of Cs‐oleate injection.^[^ ^116^ ^]^ However, the prepared Cs_2_AgBiX_6_ (X = Cl, Br) nanocrystals exhibited weak and broad PL emission as well as extremely low *PLQY*. Subsequently, Lamba et al.^[^ ^124^ ^]^ and Wang et al.^[^ ^125^ ^]^ found that the Cs_2_NaBiX_6_ (X = Cl, Br) nanocrystals were successfully synthesized by this method, respectively. Typically, CH_3_COOCs, CH_3_COONa, (CH_3_COO)_3_Bi, OA, and OLA were dissolved in ODE. Then, the TMSCl or TMSBr was swiftly injected at 140 °C under N_2_ atmosphere to obtained Cs_2_NaBiX_6_ (X = Cl, Br) nanocrystals, as routed in Equation (5):

(5) $$\begin{array}{ccc} & & 2CH_{3}\mathrm{COOCs}+{\left(CH_{3}\mathrm{COO}\right)}_{3}\mathrm{Bi}+CH_{3}\mathrm{COONa}+6{\left(CH_{3}\right)}_{3}\\ & & \;\times \;\mathrm{SiX}\;\;\overset{140{\;}^{\circ }C,\;N_{2},\;\mathrm{OA},\;\mathrm{OLA}}{\underset{\;}{\to }}Cs_{2}\mathrm{NaBi}X_{6}\mathrm{nanocrystals}\;\left(X=\mathrm{Cl},\mathrm{Br}\right) \end{array}$$

The Cs_2_NaBiCl_6_ nanocrystals showed the PL emission at 375 nm and *PLQY* of 1.7%. Furthermore, Lee and co‐workers^[^ ^126^ ^]^ observed that the cuboctahedral and cuboidal shape of Cs_2_NaBiX_6_ (X = Cl, Br) nanocrystals could be prepared by adjusting the reaction temperature. In addition, the cubic‐shaped Cs_2_AgInCl_6_ nanocrystals were also synthesized by injecting the halide source BzCl into the mixture solution of Cs‐oleate, CH_3_COOAg, (CH_3_COO)_3_In, and diphenyl ether at 105 °C and N_2_ atmosphere.^[^ ^127^ ^]^ The results indicated that the Cs_2_AgInCl_6_ nanocrystals with the average edge length of 9.8 nm exhibited broad PL emission peak centered at 560 nm and *PLQY* of 1.6%. It was worth noticing that Cs_2_AgInCl_6_ and Cs_2_AgSbCl_6_ nanocrystals could be prepared according to this specific pathway without N_2_ atmosphere.^[^ ^128^ ^]^ These facile routes are beneficial to further promote the development of lead‐free HDP nanocrystals with different elements. For instance, Cs_4_CuSb_2_Cl_12_ layered HDP nanocrystals with spherical like shape and average diameter of 12.5 nm were prepared via modified hot‐injection method.^[^ ^129^ ^]^

Based on a modified hot‐injection technique, Han group^[^ ^130^ ^]^ further developed the variable temperature hot injection method. In this approach, the reaction temperature would continue elevate to obtain nanocrystals after swiftly injecting halide precursor at a specific temperature. The cube‐shaped Cs_2_NaInCl_6_ nanocrystals with an edge length of 12.5 nm were obtained by swiftly injecting TMSCl at 165 °C and then continued to increase reaction temperature to 175 °C. This method was also suitable for the synthesis of other HDP nanocrystals, such as Cs_2_AgBiCl_6_, Cs_2_AgInCl_6_, Cs_2_AgSbCl_6_,^[^ ^130^ ^]^ and Cs_2_CuSbCl_6_,^[^ ^131^ ^]^ which would be in favor of enhancing the crystalline and obtaining single pure phase.

#### 3.1.2 Antisolvent Recrystallization Method

Generally, the hot injection technique involves relatively high reaction temperature, uncontrollable swift injection, and inert gas protection, which restrains their large‐scale production. To resolve the above‐mentioned problems, room temperature antisolvent recrystallization method is proposed to prepare lead‐free HDP nanocrystals, in which the precursor salts were first dissolved in good solvent, followed by dropping the above solution into the mixture of poor solvent and organic ligands.^[^ ^132^, ^133^ ^]^ The good solvents include dimethylformamide, or dimethylsulfoxide (DMSO) and the poor solvents are toluene hexane, or isopropanol. The precursor salts are CsX, B^+^X (B^+^ = Na, Ag), or B^3+^X_3_ (B^3+^ = Bi, In, and Sb, X = Cl, Br, and I). When a small quantity of good solvents and abundant poor solvents are mixed, an instantaneous supersaturation and immediately recrystallization of HDP nanocrystals will achieve (Figure 4b).^[^ ^134^ ^]^ However, the nucleation and growth stages of HDP nanocrystals in the antisolvent recrystallization process cannot be separated.^[^ ^135^ ^]^

The preparation of Cs_2_AgBiX_6_ (X = Cl, Br, and I) HDP nanocrystals via antisolvent recrystallization approach could date back to 2018 by Yang et al.^[^ ^52^ ^]^ The CsX, AgX, and BiX_3_ salts with molar ratio of 2:1:1 were dissolved in DMSO, and then the resulting solutions were dropped into the mixture of isopropanol and OA. They found that the quasi‐spherical shape Cs_2_AgBiBr_6_ nanocrystals with an average diameter of 5.0 nm could be obtained. Moreover, the emission wavelength of Cs_2_AgBiX_6_ (X = Cl, Br, and I) nanocrystals could adjust at 395 to 500 nm. Interesting, it can find that PL intensity of HDP nanocrystals synthesized via antisolvent recrystallization approach is significantly higher than corresponding hot injection method.^[^ ^51^ ^]^ The *PLQY* of Cs_2_AgBiCl_6_ nanocrystals was boosted to 6.7%. Subsequently, they further reported the synthesis of Cs_2_AgIn*_x_*Bi_1−_ *_x_*Cl_6_ (0 ≤ *x* ≤ 0.9) and Cs_2_AgSb_1−_ *_y_*Bi*_y_*X_6_ (X = Br, Cl; 0 ≤ *y* ≤ 1) nanocrystals by this method.^[^ ^121^, ^136^ ^]^ In 2019, Lv and co‐worker^[^ ^137^ ^]^ adopted the antisolvent recrystallization method to synthesize Cs_2_AgSbX_6_ (X = Cl, Br, and I) nanocrystals. The precursor solution could be formed after dissolving CsX, AgX, and SbX_3_ in DMSO, and then dropped into a mixture solution of OA and ethyl acetate under vigorous stirring. In this work, the spherical shape Cs_2_AgSbCl_6_ nanocrystals with average size of 4.65 nm exhibited 409 nm PL emission peak and *PLQY* of 31.33% as well as an excellent air stability. The key factor for antisolvent recrystallization approach is the amount of ligand (OA), which plays an important role in controlling the crystallization and improving PL performance of the HDP. The role of ligand will be discussed in Section 3.2.

#### 3.1.3 Halide Ion Exchange Reactions and Other Synthesis Methods

Besides, many synthesis methods were proposed to prepare new HDP nanocrystals, such as anion exchange, ultrasonic irradiation, and ultrasonic exfoliation method. Creutz et al.^[^ ^51^ ^]^ first performed post‐synthetic anion‐exchange reaction using TMSBr or TMSI to synthesize Cs_2_AgBiX_6_ (X = Br, I) nanocrystals (Figure 4c). The size and shape of the parent Cs_2_AgBiCl_6_ nanocrystals could be retained after the exchange reaction, while their composition was successfully tailored in a desired range. Koyanagi et al.^[^ ^138^ ^]^ reported an ultrasonic irradiation method to produce Cs_2_SnI_6_ nanocrystals by forming CsI reverse micelles emulsion under sonication with OA and OLA, followed by reacting with the mixture consisting of SnI_4_, OA, OLA, and ethanol. The final product exhibited larger size about 200 nm. In addition, single layered Cs_4_CuSb_2_Cl_12_ nanocrystals with a uniform size of 3 nm were first successfully prepared in 2019 by ultrasonic exfoliation method.^[^ ^139^ ^]^ In short, the synthesized bulk microcrystals by liquid‐phase solution were dispersed into solvent, and then ultrasonicated and centrifuged to obtain the final product (Figure 4d). Unfortunately, the PL property of Cs_4_CuSb_2_Cl_12_ nanocrystals was not observed.^[^ ^129^, ^139^ ^]^

Overall, the hot injection technique has reached maturity to produce nearly monodisperse HDP nanocrystals, and the morphology of nanocrystals (spherical quantum dots, nanorods, nanowires, nanobelts, nanoplatelets, and cuboctahedral) can be adjusted by changing reaction temperature and time. And this route can provide a synthetic strategy for lead‐free HDP nanocrystals constituted from different elements. However, hot injection route was commonly applied at air‐free atmosphere and the resultant products exhibited low *PLQY* owing to surface defects. These problems can be overcome by performing antisolvent recrystallization method, which can easily be used to produce HDP nanocrystals with high *PLQY*. Unfortunately, many HDPs with excellent optical properties such as Cs_2_CuInX_6_, Cs_2_InBiX_6_ by theoretical calculation are not synthesized via current synthesis strategies due to their poor stability. Hence, the synthesis strategies of lead‐free HDP nanocrystals need further devolvement.

### 3.2 Ligand Strategies

The ligand chemistry has been widely applied in preparing colloidal perovskite nanocrystals.^[^ ^140^, ^141^ ^]^ Generally, the surface ligand plays an important role for regulating the nucleation and growth process, stability, and optoelectronic properties of nanocrystals. In this section, the recent progress of ligand strategies in the HDP nanocrystals field was summarized and some examples were provided to understand the role of ligand strategies in the construction of HDP nanocrystals.

**Figure 5:**
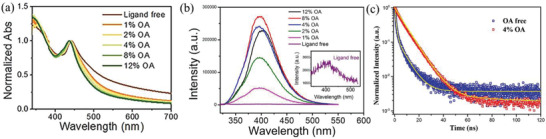
a) Steady‐state absorption spectra and b) PL spectra of Cs_2_AgBiX_6_ (X = Cl, Br) nanocrystals with different amount OA capped. Reproduced with permission.^[^ ^52^ ^]^ Copyright 2018, Wiley‐VCH. c) Time‐resolved PL kinetics of Cs_2_AgSbCl_6_ nanocrystals without and with 4% OA. Reproduced with permission.^[^ ^137^ ^]^ Copyright 2018, The Royal Society of Chemistry.

The OA and OLA are common long hydrocarbon chains ligands, which can control the nucleation and growth process of crystals and modulate the morphology of HDP nanocrystals. Xu et al.^[^ ^142^ ^]^ successfully synthesized Cs_2_SnI_6_ nanocrystals and nanoplatelets by altering different ligands. They pointed out that 3D Cs_2_SnI_6_ nanocrystals were obtained by using OA as ligand, while 2D nanoplatelets were formed using OA and organic amine as ligands. The evolution of morphology was attributed to the attachment of organic amine on the surface of perovskites, which limited the crystal growth in the attachment direction. In addition, many groups studied the role of OA and OLA on the formation of colloidal HDP nanocrystals systematically. Zhou and co‐workers^[^ ^120^ ^]^ synthesized monodisperse Cs_2_AgBiBr_6_ nanocrystals with cubic shape under the co‐reaction of OA and OLA. They observed that OLA could enhance solubility of BiBr_3_ in ODE by complexing for Bi^3+^ ions, while OA ligand could suppress the growth of crystal. Furthermore, Liu et al.^[^ ^122^ ^]^ found that large amounts of OLA easily led to the reduction from Ag^+^ to Ag^0^ for the synthesis of Bi^3+^ doped Cs_2_AgInCl_6_ nanocrystals, owing to the reduction nature of amine ligands. For Cs_2_SnI_6_ nanocrystals, Wang and co‐workers^[^ ^117^ ^]^ assumed that OLA acted as a complexing agent for Sn^4+^ ions, while OA played a role in suppressing nanocrystals growth. Intriguingly, the Cs_2_CuSbCl_6_^[^ ^131^ ^]^ and CsEuCl_3_^[^ ^44^ ^]^ nanocrystals were synthesized by hot injection method, which crafty used the reduction nature from OLA to reduce Cu^2+^ and Eu^3+^ to Cu^+^ and Eu^2+^ as reaction precursor, respectively. Another critical role of organic ligands is passivating the surface defects of HDP nanocrystals by surface capping, which could enhance radiative recombination rates. For instance, Yang et al.^[^ ^52^ ^]^ proposed that the photoelectric properties of Cs_2_AgBiX_6_ (X = Cl, Br) nanocrystals could be improved by OA capping. They found that the absorption tail of Cs_2_AgBiBr_6_ nanocrystals could be effectively suppressed with increasing the OA amounts (**Figure** **5**a) and the PL intensity showed 100 times enhancement with 8% of OA addition compare with ligand free Cs_2_AgBiCl_6_ nanocrystals (Figure 5b). Subsequently, Lv et al.^[^ ^137^ ^]^ reported that the PL intensity was significantly boosted and *PLQY* was evaluated from 4% to 31.33% after modification of Cs_2_AgSbCl_6_ quantum dots with OA ligand. The carrier lifetime of Cs_2_AgSbCl_6_ quantum dots was also enhanced from 2.16 to 7.93 ns (Figure 5c). However, other common surfactants (such as octylamine, oleylamine, and tri‐n‐octylphosphine) do not obviously improve PL property of HDP nanocrystals in antisolvent recrystallization process.^[^ ^52^, ^137^ ^]^

In summary, the OA and OLA as ligands can not only control the synthesis process but also tune the morphology of HDP nanocrystals. Especially, the photoelectric properties of HDP nanocrystals can be obviously boosted by OA capped alone. The ligand strategies provide a new path for obtaining high quality HDP nanocrystals. However, the surface passivation mechanism of capping ligands still needs to be further studied, which will be significantly instructive for boosting efficiency and stability of lead‐free HDP nanocrystals and devices.

### 3.3 Doping/Alloying Strategies

**Figure 6:**
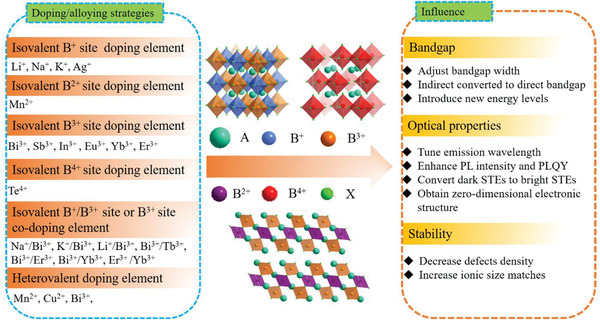
Effects of metal doping/alloying strategies on HDP nanocrystals properties

To boost or modulate photoelectric properties of lead‐free HDP nanocrystals, the metal doping/alloying strategies are widely employed.^[^ ^143^, ^144^, ^145^ ^]^ According to the substitution possibilities of the elements existed in the A_2_B^+^B^3+^X_6_, A_2_B^4+^X_6_, and A_4_B^2+^B^3+^_2_X_12_ HDP structure, we simply classify metal doping/alloying strategies of HDP nanocrystals reported in the literature into the isovalent “B^+^‐site”, “B^2+^‐site”, “B^3+^‐ site”, and “B^4+^‐ site” doping/alloying and heterovalent B‐site doping/alloying. The different valence states of B‐site metals in HDP structure provide more possibility of doping/alloying, generating fascinating photoelectricity performance for desired HDP materials. In this section, recent progress on metal doping/alloying strategies with different dopants in HDP structure was systematically summarized and their effects on the structure, bandgap, PL properties, and stability of HDP nanocrystals were discussed, as shown in **Figure** **6** and **Table** **3**.

**Table 3:** The metal doping/alloying in HDP nanocrystals and their excitation, emission, and *PLQY*

Nanocrystals	Dopants	Synthesis method	Ligands	Excitation wavelength	Emission wavelength	*PLQY*	Ref.
Cs_2_AgBiCl_6_	Na^+^	Modified hot‐injection	OA, OLA	325–350 nm	455–800nm	<1%	^[^ ^124^ ^]^
Cs_2_NaBiCl_6_	Ag^+^	Modified hot‐injection	OA, OLA	325 nm	455–800nm	20%	^[^ ^146^ ^]^
Cs_2_NaInCl_6_	Ag^+^	Variable temperature hot‐injection	OA, OLA	254 nm	400–750 nm	31.1%	^[^ ^130^ ^]^
Cs_2_AgBiCl_6_	In^3+^	Antisolvent recrystallization	OA	365 nm	395 and 570 nm	36.6% and 2%	^[^ ^136^ ^]^
Cs_2_AgInCl_6_	Bi^3+^	Hot‐injection	OA, OLA	368 nm	580 nm	11.4%	^[^ ^122^ ^]^
Cs_2_AgInCl_6_	Yb^3+^	Modified hot‐injection	OA, OLA	300 nm	996 nm	3.6%	^[^ ^153^ ^]^
Cs_2_AgInCl_6_	Er^3+^	Modified hot‐injection	OA, OLA	300 nm	1537 nm	0.05%	^[^ ^153^ ^]^
Cs_2_AgInCl_6_	Yb^3+^ + Er^3+^	Modified hot‐injection	OA, OLA	300 nm	996 and 1537 nm	0.2% and 0.02%	^[^ ^153^ ^]^
Cs_2_NaBiCl_6_	Eu^3+^	Modified hot‐injection	OA, OLA	300 nm	591, 615, 652, and 700 nm	3.3%	^[^ ^146^ ^]^
Cs_2_AgInCl_6_	Bi^3+^ + Tb^3+^	Hot‐injection	OA, OLA	368 nm	490, 550, and 620 nm	6.6≈10.1%	^[^ ^156^ ^]^
Cs_2_AgInCl_6_	Na^+^ + Bi^3+^	Antisolvent recrystallization	OA	370nm	400–700 nm at around 559 nm	64%	^[^ ^155^ ^]^
Cs_2_AgInCl_6_	K^+^ + Bi^3+^	Modified hot‐injection	OA, OLA	365 nm	390–710 nm	14.3%	^[^ ^113^ ^]^
Cs_2_AgInCl_6_	Li^+^ + Bi^3+^	Modified hot‐injection	OA, OLA	365 nm	390–710 nm	11.1%	^[^ ^113^ ^]^
Cs_2_AgBiCl_6_	Mn^2+^	Modified hot‐injection	OA, OLA	365 nm	600 and 680 nm	<1%	^[^ ^158^ ^]^
Cs_2_AgInCl_6_	Mn^2+^	Modified hot‐injection	OA, OLA	290 nm	620 nm	16%	^[^ ^127^ ^]^
Cs_2_NaBiCl_6_	Mn^2+^	Modified hot‐injection	OA, OLA	354 nm	500–700 nm at around 585 nm	3.9%	^[^ ^146^ ^]^
Cs_2_NaBiCl_6_	Mn^2+^ + In^3+^	Variable temperature hot injection	OA, OLA	‐	614 nm	44.6%	^[^ ^159^ ^]^
Cs_2_SnCl_6_	Sb^3+^	Hot‐injection	OA, OLA	365 nm	438 and 615 nm	8.25%	^[^ ^119^ ^]^
Cs_2_SnCl_6_	Mn^2+^	Hot‐injection	OA, OLA	333 nm	628 nm	–	^[^ ^164^ ^]^
Cs_2_NaInCl_6_	Sb^3+^ + Mn^2+^	Hot‐injection	OA, OLA	300‐360 nm	455 and 622 nm	10–24%	^[^ ^160^ ^]^

**Table 4:** Air storage, thermal, water, and UV light stability of HDPs

Materials	Air storage stability	Thermal stability	Water stability	UV light stability	Ref.
Cs_2_AgInCl_6_ nanocrystals	Structure stability under air for one week	Thermally stable up to ≈500 °C	–	–	^[^ ^127^ ^]^
Cs_2_AgSbCl_6_ nanocrystals	Phase stability, 90% of initial PL intensity after 6 months in 55% humidity at 25 °C and dark	–	–	–	^[^ ^137^ ^]^
Cs_2_AgBiBr_6_ nanocrystals @mesoporous silica	Phase stability after humidity of 55% for 180 days	–	–	–	^[^ ^173^ ^]^
Bi^3+^ doped Cs_2_ZrCl_6_ bulk	–	Phase stability, PL intensity unchanged after heat treatment at 100 and 150 °C for 30 min	PL intensity enhanced 115.94% after immersing 2 h by modified long alkyl chains	–	^[^ ^68^ ^]^
Sb^3+^ doped Cs_2_AgBiBr_6_ nanocrystals	PL intensity unchanged after stored in air for one month	–	–	–	^[^ ^121^ ^]^
Mn^2+^ doped Cs_2_SnCl_6_ nanocrystals	PL intensity almost unchanged after stored in air for 4 days	–	–	–	^[^ ^164^ ^]^
Yb^3+^ doped Cs_2_AgInCl_6_ microcrystals	Phase stability after 3 months in room temperature and relative humidity ≈30%	–	–	–	^[^ ^152^ ^]^
Bi^3+^ doped Cs_2_AgInCl_6_ nanocrystals	Only 10% emission decay under 75% moisture for 100 h and structure stability after storage 3 months	Approximately 20% of initial PL intensity under heated at 100 °C, 90% of initial PL intensity after 100 °C for 50 h, 80% under 4 heating/cooling cycles	–	60% of original PL intensity of under UV irradiation for 50 h	^[^ ^174^ ^]^
Bi^3+^ doped Cs_2_Ag_0.60_Na_0.40_InCl_6_ bulk	–	Almost no decay under different temperatures from 233 to 343 K or continuous heating at 150 °C for 1000 h	–		^[^ ^74^ ^]^
Sb^3+^ doped Cs_2_NaInCl_6_ bulk	–	thermally stable up to ≈600 °C	–	90% of original PL intensity after continuous UV light for 1000 h.	^[^ ^175^ ^]^
Bi^3+^ doped Cs_2_SnCl_6_ bulk	–	–	97.1% of original PL intensity after immersing 2 h	–	^[^ ^65^ ^]^
Te^4+^ doped Cs_2_SnCl_6_ bulk	–	–	Preserving 100% of the original PL intensity after 6 h soaking	–	^[^ ^67^ ^]^

#### 3.3.1 Isovalent Metal Doping/Alloying

##### Isovalent B^+^‐Site Metal Doping/Alloying

**Figure 7:**
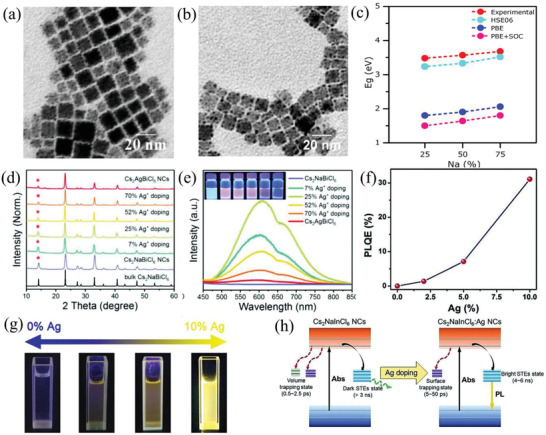
TEM images of a) Ag^+^ doped Cs_2_NaBiCl_6_ nanocrystals and b) Cs_2_AgBiCl_6_ nanocrystals. c) Bandgap from experimental and DFT calculations with different function. Reproduced with permission.^[^ ^124^ ^]^ Copyright 2019, American Chemical Society. d) XRD patterns of Ag^+^ doped Cs_2_NaBiCl_6_ nanocrystals with different doping amount of Ag^+^. e) PL spectra of the Cs_2_Na_1−_ *_x_*Ag*_x_*BiCl_6_ nanocrystals (*x* = 0, 0.07, 0.25, 0.52, 0.70, and 1), the insert is the photos of nanocrystals under UV light irradiation. Reproduced with permission.^[^ ^146^ ^]^ Copyright 2020, Wiley‐VCH. f) *PLQY* and g) photographs of Ag^+^ doped Cs_2_NaInCl_6_ nanocrystals with different doping amount of Ag^+^, h) The STEs of Cs_2_NaInCl_6_ and Ag^+^ doped Cs_2_NaInCl_6_ nanocrystals. Reproduced with permission.^[^ ^130^ ^]^ Copyright 2019, Wiley‐VCH.

Based on the adjustable component, the typical effects of metal doping/alloying on HDP nanocrystals are their tunable bandgap width and PL emission intensity. Commonly, the monovalent alkali metal (such as Li^+^, Na^+^, and K^+^) and Ag^+^ were intentionally introduced in B^+^‐site of HDP nanocrystals to tune bandgap and thereby boost the PL emission intensity. For instance, Lamba et al.^[^ ^124^ ^]^ synthesized Cs_2_(Na*_x_*Ag_1−_ *_x_*)BiCl_6_ (*x* = 0, 0.25, 0.5, 0.75, and 1) HDP nanocrystals by modified hot‐injection method. The Na^+^ doped and undoped Cs_2_AgBiCl_6_ nanocrystals showed the same cubic shape and size (**Figure** **7**a,b). Meanwhile, the emission intensities of Cs_2_Na_0.75_Ag_0.25_BiCl_6_ nanocrystals in the orange region showed 30‐fold increase compared with undoped sample due to the conversion from non‐radiative transitions to radiative transitions after Na^+^ doping. The experimental and DFT theoretical results indicated that the optical band gap increased from 3.39 to 3.82 eV with increasing the doping amount of Na^+^ from 0 to 1, owing to the contribution of Ag^+^ reduction in near VBM by incorporation of Na^+^ ion (Figure 7c). Furthermore, Yao et al.^[^ ^146^ ^]^ demonstrated that the intensity of the (111) peak decreased with an increasing Na/Ag ratio in the lattice by XRD results (Figure 7d), which confirmed the formation of the alloyed structure. The excitonic absorption energy of the Cs_2_NaBiCl_6_ nanocrystals could be tuned from 3.82 to 3.48 eV, and the *PLQY* could be significantly improved from 1.7% to 20% with increasing Ag^+^ doped content from 0 to 0.25, which exhibited bright orange‐red emission centered at 613 nm (Figure 7e). Zhu and co‐workers^[^ ^147^ ^]^ further analyzed carrier dynamics in Cs_2_Na_1−_ *_x_*Ag*_x_*BiCl_6_ nanocrystals. They observed that the Ag^+^ ions acted as centers in Na^+^ rich Cs_2_Na_1−_ *_x_*Ag*_x_*BiCl_6_ system, which could localize both holes and electrons at the band edges, resulting in efficient radiative recombination in spatially connected AgCl_6_‐BiCl_6_ octahedra. For Cs_2_Na_1−_ *_x_*Ag*_x_*BiBr_6_ system, Dai et al.^[^ ^148^ ^]^ demonstrated that Cs_2_AgBiBr_6_ and Cs_2_NaBiBr_6_ had a small lattice‐mismatch based on first principles calculations, so their alloys Cs_2_(Na*_x_*Ag_1−_ *_x_*)BiBr_6_ were highly miscible and the bandgaps could be adjusted in a wide range of 1.93 to 3.24 eV when the composition ratio *x* increased from 0 to 1. Hence, changing the Ag^+^ composition could adjust the bandgaps of the Ag^+^ rich light‐emitting centers, resulting in the tunable emission wavelength and broadband emission. In addition, Han et al.^[^ ^130^ ^]^ observed that the PL intensity and *PLQY* of Cs_2_NaInCl_6_ nanocrystals were considerably enhanced after Ag^+^ doping. The Ag^+^ doped Cs_2_NaInCl_6_ nanocrystals exhibited a broad bright yellow emission ranging from 400 to 750 nm with an emission center of 535 nm and the highest *PLQY* was 31.1% as the Ag^+^ doping ratio reached 10% (Figure 7f,g), which was ascribed to conversion from dark STEs to bright STEs (Figure 7h). Furthermore, the stability of Ag^+^ doped Cs_2_NaInCl_6_ nanocrystals was obviously boosted under air exposure for over a month compared with undoped samples. For Cs_2_CuSbCl_6_ nanocrystals, the Ag^+^ alloyed nanocrystals can change the absorption from 530 to 365 nm and optical bandgap from 1.66 to 3.10 eV, which exhibited great potential in photovoltaic applications.^[^ ^131^ ^]^

##### Isovalent B^2+^‐Site Metal Doping/Alloying

The isovalent B^2+^‐site metal doping/alloying only exists in A_4_B^2+^B^3+^_2_X_12_ layered perovskite structure. Although a few layered HDP materials were designed and synthesized, the PL properties were unsatisfactory for LEDs application. For instance, the reported Cs_4_CuSb_2_Cl_12_ nanocrystals have no photoluminescence property,^[^ ^96^, ^129^ ^]^ Cs_4_CdBi_2_Cl_12_ phosphor exhibited emission peak at 605 nm and *PLQY* of ≈4%,^[^ ^79^ ^]^ and Cs_4_MnBi_2_Cl_12_ perovskite single crystal showed orange emission with a *PLQY* of 25.7%.^[^ ^78^ ^]^ Hence, Mn^2+^ doped Cs_4_CdBi_2_Cl_12_ vacancy‐ordered double perovskites were proposed by Holzapfel et al.^[^ ^79^ ^]^ The replacement of Mn^2+^ by Cd^2+^ could obtain 0D electronic structure, resulting in an improved *PLQY* of 57% and an enhanced PL intensity. The Mn^2+^ as dopant can change electronic structure of host, which is an effective strategy to regulate fluorescence performance and stability of HDPs.

##### Isovalent B^3+^‐Site Metal Doping/Alloying

**Figure 8:**
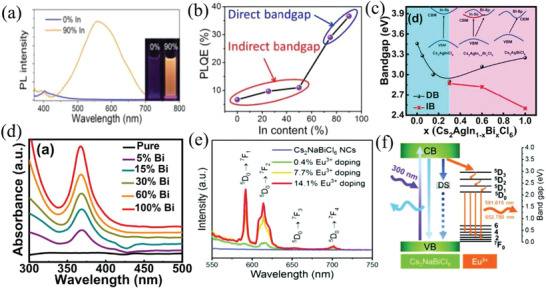
a) PL spectra and images of Cs_2_AgIn*_x_*Bi_1−_ *_x_*Cl_6_ (x = 0 and 0.9) nanocrystals. b) *PLQY* value of Cs_2_AgIn*_x_*Bi_1−_ *_x_*Cl_6_ (x = 0, 0.25, 0.5, 0.75, and 0.9) nanocrystals. Reproduced with permission.^[^ ^136^ ^]^ Copyright 2018, American Chemical Society. c) The bandgap value of Cs_2_AgIn_1−_ *_x_*Bi*_x_*Cl_6_ nanocrystals measured from Tauc plots for direct and indirect transition (DB: direct bangap, IB: indrect bandgap). d) UV–vis absorption spectra of Cs_2_AgIn_1−_ *_x_*Bi*_x_*Cl_6_ nanocrystals. Reproduced with permission.^[^ ^58^ ^]^ Copyright 2019, American Chemical Society. e) PL spectra of Eu^3+^doped Cs_2_NaBiCl_6_ nanocrystals under excitation at 300 nm. f) PL mechanism of Eu^3+^doped Cs_2_NaBiCl_6_ nanocrystals. Reproduced with permission.^[^ ^146^ ^]^ Copyright 2020, Wiley‐VCH.

The indirect band can be converted to direct band by doping trivalent transition metal.^[^ ^92^, ^149^ ^]^ Yang et al.^[^ ^136^ ^]^ synthesized Cs_2_AgIn*_x_*Bi_1−_ *_x_*Cl_6_ (*x* = 0, 0.25, 0.5, 0.75, and 0.9) nanocrystals with double‐color emission at 395 nm (violet) and 570 nm (orange) via antisolvent recrystallization method (**Figure** **8**a). The band structure of Cs_2_AgIn*_x_*Bi_1−_ *_x_*Cl_6_ nanocrystals could be tuned from the indirect bandgap (*x* = 0, 0.25, and 0.5) to direct bandgap (*x* = 0.75 and 0.9). Meanwhile, the *PLQY* of In^3+^ doped nanocrystals with 36.6% in violet region was about 5 times as much as that of undoped samples (6.7%) (Figure 8b). Furthermore, Manna et al.^[^ ^150^ ^]^ studied the band structure of Bi^3+^ doped Cs_2_AgInCl_6_ nanocrystals in detail. They observed that the width of bandgap reduced and the VBM composed of hybridization between the Ag 4d and Cl 3p transformed into Bi 6p orbital with increasing substitution ratio of Bi^3+^ (Figure 8c). The highest *PLQY* of Cs_2_AgIn_1−_ *_x_*Bi*_x_*Cl_6_ nanocrystals was about 10% under 5% Bi^3+^ doping and the intensity of the absorption enhanced with increasing the doping concentration of Bi^3+^ (Figure 8d).^[^ ^58^ ^]^ Furthermore, the decrease of the hole effective mass with Bi^3+^ incorporation in the alloys Cs_2_AgInCl_6_ could improve the carrier mobility, making it promising for white light emission applications. In addition, Li et al.^[^ ^151^ ^]^ revealed that, as deep electron traps with low formation energy, In_Ag_ was the intrinsic defect in Cs_2_AgInCl_6_, which could be suppressed by low doping concentration of Bi^3+^. Luo et al.^[^ ^74^ ^]^ demonstrated that Bi^3+^ doping Cs_2_Na*_x_*Ag_1−_ *_x_*InCl_6_ could further diminish defects, resulting in the enhancement of *PLQY* to 86%.

In addition, the PL or the appearance of a new spectral stems from the energy transfer between host energy levels and guest energy levels by doping lanthanide ions. For instance, doping Yb^3+^ into the C_2_AgInCl_6_ host lattice created near IR emission at 996 nm. The energy from absorption of light transferred from the nanocrystals to excite the Yb^3+^ ions and then the f–f de‐excitation from ^2^F_5/2_ → ^2^F_7/2_ emitting NIR light.^[^ ^152^ ^]^ The *PLQY* of Yb^3+^ doped C_2_AgInCl_6_ nanocrystals could be elevated from 1.8% to 3.6% with the increase of Yb^3+^ doping amount from 0.6% to 0.9%.^[^ ^153^ ^]^ Similarly, Er^3+^ doped C_2_AgInCl_6_ nanocrystals were reported. The *PLQY* of Er^3+^ doped nanocrystals with emission peak at 1537 nm was about 0.05%. For Eu^3+^ doping, the exciton energy transferred from Cs_2_NaBiCl_6_ nanocrystals host to higher energy level of Eu^3+^ ion and then transmitted to ^5^D_0_ → ^7^F_J_ (J = 1, 2, 3, and 4) by the nonradiative relaxation (Figure 8f).^[^ ^146^ ^]^ Thus, the Eu^3+^ doped nanocrystals exhibited orange‐red emissions and the *PLQY* values were around 3% (Figure 8e).

##### Isovalent B^4+^‐Site Metal Doping/Alloying

Other than the widely studied isovalent B^+^ site doped and B^3+^ site doped systems, a few other quadrivalence cations have also been tested in HDPs. The Te^4+^ cations as dopants to boost PL property and stability of Cs_2_SnCl_6_ HDPs have been introduced by hydrothermal method.^[^ ^67^ ^]^ The TeCl_4_·4H_2_O were added to the other precursors during the synthesis of Cs_2_SnCl_6_ solid‐solution materials, and it was found that the Te^4+^ was successfully incorporated into the Cs_2_SnCl_6_. The formation of [TeCl_6_]^2−^ octahedron in the Cs_2_SnCl_6_ lattice structure enhanced the Jahn–Teller‐like STEs. The solid‐solution materials exhibited bright yellow green luminescence at 580 nm with *PLQY* of 95.4%, which had great potential for lighting applications. However, the nanocrystals about isovalent B^4+^‐site metal doping/alloying HDPs have never been reported.

##### Isovalent B^+^/B^3+^‐Site and B^3+^‐Site Metal Co‐Doping/Alloying

The impacts of B‐site metal doping/alloying in HDP materials have complexity and diversity, because the atomic orbitals and site occupation of B^+^ and B^3+^ cations have an enormous effect on the bandgap and excitons radiation channel. Although many groups reported that B^+^‐site or B^3+^‐site doping could improve or tune optical properties of the HDP hosts, they were still unsatisfactory for LEDs application. Hence, isovalent B^+^/B^3+^‐site and B^3+^‐site metal co‐doping/alloying of HDP nanocrystals were studied. Locardi et al.^[^ ^154^ ^]^ first reported Bi^3+^ doped Cs_2_Ag_1−_ *_x_*Na*_x_*InCl_6_ nanocrystals via modified hot injection method. They found that the incorporation of Bi^3+^ could form new BiCl_6_ states below CBM, whereas the Na^+^ doping promoted localization of AgCl_6_ energy levels above the VBM. The bright PL emission derived from recombining via BiCl_6_ → AgCl_6_ transition. Further, Tang group^[^ ^155^ ^]^ obtained the Cs_2_Ag_1−_ *_x_*Na*_x_*In_1−_ *_y_*Bi*_y_*Cl_6_ nanocrystals with high *PLQY* of 64% through Na^+^/Bi^3+^ ions co‐doping and ligand passivation. Incorporation of Na^+^ and Bi^3+^ cations into Cs_2_AgInCl_6_ host could break of parity‐forbidden transition, which possessed longest lifetime of 6.53 µs. Cong et al.^[^ ^113^ ^]^ investigated STE effects by alloying K^+^ or Li^+^ ions and Bi^3+^ ions in Cs_2_AgInCl_6_ nanocrystals. The Femtosecond transient absorption and DFT calculations indicated that the broadband white‐light emission originated from the further suppressing non‐radiative processes by the STEs in the direct bandgap structure. The Cs_2_K*_x_*Ag_1−_ *_x_*In_0.99_Bi_0.01_Cl_6_ and Cs_2_Li*_y_*Ag_1−_ *_y_*In_0.99_Bi_0.01_Cl_6_ nanocrystals showed Commission Internationale de I'Eclairage (CIE) of (0.37, 0.41) and (0.37, 0.42), which had promising application in “warm” white LEDs (WLEDs). For isovalent B^3+^‐site metal co‐doping/alloying of HDPs, the Yb^3+^‐Er^3+^ co‐doped Cs_2_AgInCl_6_ nanocrystals were reported, which exhibited double emission with 996 and 1537 nm.^[^ ^153^ ^]^ Unfortunately, the *PLQY* and PL intensity of Yb^3+^‐Er^3+^ co‐doped nanocrystals were not significantly improved. Interestingly, Liu et al.^[^ ^156^ ^]^ reported that Bi^3+^ and Tb^3+^ co‐doped C_2_AgInCl_6_ nanocrystals can also obtain new sharp emission peaks located at around 490, 550, and 620 nm, corresponding to the intrinsic transition of Tb^3+^ ions ^5^D_4_ → ^7^F_6_, ^5^D_4_ → ^7^F_5_, and ^5^D_4_ → ^7^F_3_, besides for obtained broad emissions derived from STEs. However, the Bi^3+^ and Tb^3+^ co‐doped nanocrystals have no significant improvement in PL intensity compared with Bi^3+^ doped C_2_AgInCl_6_ nanocrystals. For another, the *PLQY* of Tb^3+^‐Bi^3+^ co‐doping nanocrystals decreased from 10.1% to 6.6% with the increase of Tb^3+^ doping content from 0 to 20.1 mol%.^[^ ^122^, ^156^ ^]^ Further, Bi^3+^ and lanthanide ions have been proposed and successfully co‐doped into the lattices of HDPs. For instance, the Yb^3+^‐Bi^3+^ and Er^3+^‐Bi^3+^ co‐doped C_2_AgInCl_6_ HDPs with near IR emission were reported, in which Bi^3+^‐Er^3+^ co‐doped hosts exhibited ≈45 times higher emission intensity compared to the Er^3+^ doped Cs_2_AgInCl_6_.^[^ ^157^ ^]^ Wang et al. found that the *PLQY* of Cs_2_Ag_0.4_Na_0.6_InCl_6_ could be boosted from 89.9% to 98.6% and 98.4% via Bi^3+^‐Ce^3+^ and Bi^3+^‐Ni^3+^ co‐doped, respectively, which were the highest value in the reported HDP materials and exhibited a great potential in solid‐state lighting.^[^ ^76^ ^]^

#### 3.3.2 Heterovalent Metal Doping/Alloying

Apart from the mentioned isovalent metal cations doping/alloying for HDP nanocrystals, several other heterovalent metal ions such as Mn^2+^ were also frequently reported for improving photoelectric performance. Chen et al.^[^ ^158^ ^]^ first synthesized Mn^2+^ doped Cs_2_AgBiCl_6_ nanocrystals via modified hot‐injection method. They demonstrated that the PL lifetime of Mn^2+^ doped Cs_2_AgBiCl_6_ nanocrystals was 0.60 ms, which was well consistent with the assigned spin‐forbidden transition of the Mn^2+^ ion centers. Similarly, Locardi and co‐workers^[^ ^127^ ^]^ reported that Mn^2+^ doped Cs_2_AgInCl_6_ nanocrystals exhibited a bright red PL emission centered at ≈620 nm and the *PLQY* was as high as ≈16% owing to the ^4^T_1_ → ^6^A_1_ transition of Mn^2+^ dopants, while undoped Cs_2_AgInCl_6_ nanocrystals showed a weak broad emission at 560 nm. Subsequently, Han et al.^[^ ^159^ ^]^ synthesized Mn^2+^ doped Cs_2_NaBi_1−_ *_x_*In*_x_*Cl_6_ nanocrystals via variable temperature hot injection, which could obtain bright orange red emission in the range from 583 to 614 nm with increasing In^3+^ content, and the highest *PLQY* was 44.6%. Further, Liu and co‐workers^[^ ^160^ ^]^ found that Mn^2+^ doped Cs_2_NaIn_1−_ *_x_*Sb*_x_*Cl_6_ nanocrystals showed dual emission at 455 and 622 nm. However, the *PLQY* of co‐doped Cs_2_NaInCl_6_ nanocrystals gradually decreased from 24% to 10% with the increase of Mn^2+^ content, which could be attributed to the increased volume defect density caused by the incorporation of Mn^2+^ dopant. In addition, Cu^2+^ can also be used as heterovalent metal dopant in HDP materials. The Cu^2+^ as dopant was successfully incorporated into the lattice of Cs_2_AgInCl_6_ nanocrystals, which could reduce bandgap from 3.60 to 2.19 eV with the increase of the Cu^2+^ doping content from 0% to 3.4% due to the contribution of Cu‐3d orbitals in the VBM.^[^ ^161^ ^]^ Similarly, Cu^2+^ doped Cs_2_AgSbCl_6_ HDPs can significantly change bandgap, in which the bandgaps decreased from 2.6 to 1.02 eV after Cu^2+^ doping amount increased from 0 to 0.1.^[^ ^162^ ^]^ However, the PL property of Cu^2+^ doping HDPs have not obviously improved.

The heterovalent ions of Sb^3+^ ions doped Cs_2_SnCl_6_ vacancy‐ordered HDP nanocrystals were reported by Jing et al.^[^ ^119^ ^]^ They observed that the Sb^3+^ doped Cs_2_SnCl_6_ nanocrystals possessed dual emission at 438 nm and 615 nm with *PLQY* of 8.25%, while undoped Cs_2_SnCl_6_ nanocrystals exhibited blue emission at 438 nm with *PLQY* of 4.37%. The enhancement of PL performance is realized due to triplet STEs, which is attributed to the ^3^P_n_−^1^S_0_ transitions (*n* = 0, 1, and 2). And the stability of nanocrystals was improved by decreasing surface or lattice defects under Sb^3+^ doping. Subsequently, the aliovalent Bi^3+^ doped Cs_2_ZrCl_6_ nanocrystals were reported.^[^ ^163^ ^]^ The Bi^3+^ doped Cs_2_ZrCl_6_ nanocrystals showed two additional PL excitation peak at 362 and 310 nm, while the PL excitation peak of Cs_2_ZrCl_6_ nanocrystals was located at 245 nm. Hence, the Bi^3+^ doped nanocrystals exhibited bright bluish emission at 449 nm under 365 UV light, while undoped Cs_2_ZrCl_6_ nanocrystals only exhibited negligible PL emission, which indicated that Bi^3+^ doping could adjust PL excitation peak. In addition, Mn^2+^ doped Cs_2_SnCl_6_ nanocrystals were also successfully prepared via hot injection.^[^ ^164^ ^]^ Although the prepared nanocrystals exhibited double emission peak after Mn^2+^ doping, the PL properties of nanocrystals had not been remarkably improved.

In summary, the metal cation doping/alloying strategies are effective pathways for modulating photoelectric performance of lead‐free HDPs, which can realize the variable bandgap, adjustable electronic structure, enhanced PL intensity, and tunable emission peak and PL excitation peak. Although the HDP bulk materials prepared via metal cation doping are almost the same *PLQY* as lead halide perovskite nanocrystals,^[^ ^6^, ^67^, ^76^ ^]^ the PL properties of lead‐free HDPs nanocrystals still are unsatisfactory for practical commercialization for future solid‐state lightings and displays. Hence, boosting PL properties of HDP nanocrystals by metal doping/alloying strategies still needs further study.

## 4 Stability of Halide Double Perovskite Nanocrystals

The stability issue of halide perovskite materials is an ongoing challenge under continuous humidity, oxygen, heating, or irradiation condition due to its ionic nature,^[^ ^85^ ^]^ which seriously affects the properties of nanocrystals and LED devices. Despite most HDP materials present excellent stability compared with lead halide perovskite nanocrystals, while the lead‐free iodide double perovskites or HDPs with both lone‐pair states in B^+^/B^3+^ sites still suffer degeneration due to low thermodynamics stability. These HDP nanocrystals may decompose or be difficult to prepare in oxygen environment. The bottleneck of stability has been restricting the development of perovskite nanocrystals and LED devices. Hence, how to synthesize high stability HDP materials or improve their stability is need to be solved urgently.

The sources of the instability of HDPs can be analyzed in theory. Usually, the structural stability of HDPs can be evaluated by considering two empirical quantities, that is, the geometrical tolerance factor (Goldschmidt factor *t*, Equation (6))^[^ ^165^ ^]^ and the cation/anion radius ratios (octahedral factor *μ*, Equation (7)),^[^ ^166^ ^]^ where *r*_A_, *r*_B_, and *r_x_* are the radii of corresponding ions. For A_2_B^+^B^3+^X_6_ HDP structure, the effective *t*_eff_ and *μ*_eff_ can be defined in Equations (8) and (9):

(6) $$t\;=\frac{r_{A\;}+\;r_{X}}{\sqrt{2}\;(r_{B}\;+\;r_{X})}\;$$

(7) $$\mu \;=\frac{r_{B}}{r_{X}}\;$$

(8) $$t_{\mathrm{eff}}=\frac{r_{A\;}+\;r_{X}}{\sqrt{2}\left\{\frac{\left(r_{B^{+}\;}+\;r_{B^{3+}}\right)}{2}\;+\;r_{x}\right\}}\;$$

(9) $$\mu_{\mathrm{eff}}=\frac{r_{B^{+}}\;+\;r_{B^{3+}}}{2r_{x}}\;$$

In theoretical conditions, the formability of 3D HDPs requires 0.44 < *μ* <0.90 and 0.81 < *t* < 1.11.^[^ ^167^, ^168^ ^]^ When *t* is close to 1, the HDPs can maintain a high symmetry and stability. For example, the *μ*_eff_ *^+^*_,_ *μ*_eff_^3^ *^+^*,and *t*_eff_ values of Cs_2_AgInCl_6_ are 0.635, 0.442, and 0.94, respectively, which shows cubic $\left(\mathrm{Fm}\bar{3}m\right)$ perovskite structure.^[^ ^108^, ^169^ ^]^ In addition, the *μ*_eff_ *^+^*_,_ *μ*_eff_^3^ *^+^*,and *t*_eff_ values of Cs_2_AgInBr_6_ are 0.587, 0.408, and 0.93, respectively, while those values of Cs_2_AgInI_6_ are 0.523, 0.364, and 0.91,^[^ ^169^ ^]^ respectively. It can find the *μ*_eff_^3^ *^+^* values of Cs_2_AgInBr_6_ and Cs_2_AgInI_6_ are obviously low ideal range, which may lead to instability of iodide and bromide‐containing double perovskites. These prediction models can provide more information to evaluate the stability of most HDPs, which are usually considered as necessary but not sufficient condition. On the other hand, the thermodynamic stability of HDPs can be evaluated by calculating their decomposition energies (∆*H*_d_) with respect to possible pathways using first principles.^[^ ^170^ ^]^ For instance, Xiao et al.^[^ ^169^ ^]^ found that only Cs_2_AgInCl_6_ show positive ∆*H*_d_ value, while ∆*H*_d_ of Cs_2_AgInBr_6_ and Cs_2_AgInI_6_ exhibit negative values. These results indicated that the thermodynamic stability of Cs_2_AgInBr_6_ and Cs_2_AgInI_6_ are poorer than that of chloride counterparts. In summary, the goldschmidt factor *t*, octahedral factor *μ* and decomposition enthalpies can be performed to assess their stability,^[^ ^171^, ^172^ ^]^ which give insights into the HDP stability and develop strategies for making more efficient LED devices.

**Figure 9:**
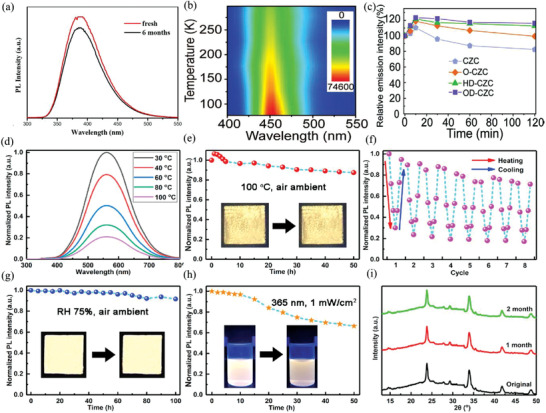
a) PL spectra of Cs_2_AgSbCl_6_ nanocrystals stored for 6 months. Reproduced with permission.^[^ ^137^ ^]^ Copyright 2018, The Royal Society of Chemistry. b) Pseudocolor map of the temperature dependent PL spectra of Bi^3+^ doped Cs_2_ZrCl_6_ at low temperature. c) Relative PL intensity of CZC (Bi^3+^ doped Cs_2_ZrCl_6_), O‐CZC (CZC surface‐modified by trimethoxy(octyl)silane), HD‐CZC (CZC surface‐modified by hexadecyltrimethoxysilane), and OD‐CZC (CZC surface‐modified by trimethoxy(octadecyl)silane) after soaked in deionized water with different times. Reproduced with permission.^[^ ^68^ ^]^ Copyright 2020, Wiley‐VCH. Normalized PL spectra of Bi^3+^ doped Cs_2_AgInCl_6_ with d) different temperatures from 30 to 100 °C, e) different time under continuous heating at 100 °C, f) different heating/cooling cycling at the temperature from 30 to 100 °C, g) different time under a relative humidity of 75%, h) different time under continuous UV light irradiation, and the insets are the photos of the sample before and after the test. i) XRD patterns of the Bi^3+^ doped Cs_2_AgInCl_6_ nanocrystals with different storing time. Reproduced with permission.^[^ ^174^ ^]^ Copyright 2020, Elsevier.

In experimentally, the chloride double perovskite nanocrystals show superior stability under different environmental conditions (**Table** 4), owing to the particular structural features and intermolecular interaction. For instance, Locardi et al.^[^ ^127^ ^]^ found that the crystal structure of Cs_2_AgInCl_6_ nanocrystals could be retained after exposure in air after several days. Moreover, the Cs_2_AgInCl_6_ nanocrystals possessed excellent thermally stability of up to ≈500 °C. Lv et al.^[^ ^137^ ^]^ demonstrated that Cs_2_AgSbCl_6_ nanocrystals powder exhibited outstanding air storage stability in 55% humidity at 25 °C in the dark for 6 months, whose PL intensity had retained 90% of the initial intensity and no obvious impure peaks were observed (**Figure** **9**a). Yet, the bromide and iodide chloride double perovskite nanocrystals suffer stability trouble due to its ionic nature, dissatisfactory *t* and *µ*, as well as low decomposition enthalpies. Hence, many strategies have been made to improve stability of HDP materials for the development of new perovskite nanocrystals and LEDs application. First, the nanocrystals can significantly induce phase stabilization. The low stability of Cs_2_AgBiI_6_ bulk had been noticed by the theoretical calculation due to unstable thermodynamically,^[^ ^107^ ^]^ and thus, the bulk materials or thin film of Cs_2_AgBiI_6_ were not successfully synthesized. Creutz et al.^[^ ^51^ ^]^ demonstrated that Cs_2_AgBiI_6_ nanocrystals could be obtained via an anion‐exchange method from Cs_2_AgBiBr_6_ nanocrystals. Similarly, the Cs_2_AgBiI_6_ nanocrystals were synthesized via room temperature antisolvent recrystallization method,^[^ ^52^ ^]^ indicating that nanocrystals can induce phase stabilization. Second, the coating and surface modification are mentioned for stabilizing nanocrystals. The Cs_2_AgBiBr_6_ nanocrystals possessed excellent humidity and light stability under carefully controlled chemical conditions. However, the amine ligands were used to prepare HDP nanocrystals, thereby forming metallic silver, which led to deterioration of the HDP structures. From the thermodynamic point of view, the formation of metallic silver was furthersome under bromine poor conditions. The decomposition pathways follow Equation (10):

(10) $$3Cs_{2}\mathrm{AgBiB}r_{6}\to Cs_{3}Bi_{2}Br_{9}+Cs_{3}\mathrm{BiB}r_{6}+3\mathrm{Ag}+3/2Br_{2}$$

To improve stability, the Cs_2_AgBiBr_6_ nanocrystals were placed within mesoporous silica, whose structure could be kept in the air with relative humidity of 55% for 180 days, indicating a high stability of the samples.^[^ ^173^ ^]^ The Cs_2_ZrCl_6_ exhibited excellent thermal stability after Bi^3+^ doping (**Figure** 9b), and the emission intensity almost unchanged after heating at 373 and 423 K for 30 min.^[^ ^68^ ^]^ Intriguingly, the emission intensity of Bi^3+^ doped Cs_2_ZrCl_6_ via modifying with trimethoxy(octadecyl)silane coupling agent can enhance to 115.94% of unmodified sample after immersing in water for 2 h (Figure 9c). Third, metal doping/alloying is an important strategy to improve stability of HDP materials by modulating *t*_eff_ and induce lattice strain. Yang et al.^[^ ^121^ ^]^ reported that the incorporation of Sb^3+^ into Cs_2_AgBiBr_6_ nanocrystals could bring superior stability in air for one month due to the better ionic size matches. In addition, metal doping/alloying can further boost stability of chloride double perovskite nanocrystals. For instance, the emission intensity of Mn^2+^ doped Cs_2_SnCl_6_ nanocrystals almost unchanged for 4 days in air, while the emission intensity of undoped Cs_2_SnCl_6_ nanocrystals declined markedly, indicating that the metal doping or alloying can boost stability of nanocrystals.^[^ ^164^ ^]^ Similarly, the Cs_2_AgInCl_6_ microcrystals exhibited excellent structure stability after 3 months in room temperature with relative humidity of ≈30%.^[^ ^152^ ^]^ The Cs_2_AgInCl_6_ nanocrystals/films via Bi^3+^ doping exhibited remarkable stability against heat, UV light, and environmental moisture/oxygen, which could keep ≈20% of the initial PL intensity after heating at 100 °C, 90% of the initial PL intensity at 100 °C for 50 h, 80% of the initial PL value under 4 heating/cooling cycles, only 10% emission decay under 75% moisture for 100 h, and 60% of the initial PL value under UV irradiation for 50 h as well as structure stability after storage 3 months(Figure 9d–i),^[^ ^174^ ^]^ respectively. It is worth mentioned that the chloride double perovskites also exhibit more excellent stability. For example, Luo et al.^[^ ^74^ ^]^ reported that the PL intensity of Bi^3+^ doping Cs_2_Ag_0.60_Na_0.40_InCl_6_ powders exhibited little decay when enhanced the temperature from 233 to 343 K or continuous heated at 150 °C for 1000 h, which indicated that the as‐prepared materials exhibited excellent stability without any encapsulation. The decomposition temperature of Sb^3+^ doped Cs_2_NaInCl_6_ materials (600 °C) was much higher than that of Cs_2_AgInCl_6_ (507 °C) and the PL intensity of Sb^3+^ doped Cs_2_NaInCl_6_ materials still hold nearly 90% of the original value after continuous illumination under UV light for 1000 h.^[^ ^175^ ^]^ For water stability, the Bi^3+^ doped Cs_2_SnCl_6_ can obtain 97.1% of the initial PL intensity after 120 min immersing.^[^ ^65^ ^]^ Furthermore, Te^4+^ doped Cs_2_SnCl_6_ can preserve 100% of the initial PL intensity after soaking for 360 min, which exhibited impressive water stability.^[^ ^67^ ^]^ These interesting results will inspire more outstanding work to design more stable of lead‐free HDP nanocrystals for lighting and display.

The nature of ionic migration in HDPs has been proved in recent literatures, which is accompanied phase separation issue. For instance, Tang group^[^ ^176^, ^177^ ^]^ found that Cs_2_AgBiBr_6_ showed ionic migration nature due to the major ionic migration channels from bromide vacancies, which was similar to regular mixed halide perovskites. Interestingly, a recent report thought that Cs_2_AgBiBr_6_ exhibited a unique dual‐ion‐migration phenomenon, where Ag^+^ and Br^−^ ions gradually diffused through the hole‐transporting layer in the long‐term operation due to the low formation energies of the Ag and Br vacancies,^[^ ^178^ ^]^ which was different with halide perovskites. In other words, Cs_2_AgBiBr_6_ is prone to phase separation, which results in the formation of AgBr phase due Ag ions and Br ions migration. In summary, ion migration phenomenon is easy to occur in HDPs, which can be eliminated via passivating the grain boundary to obtain high stable devices.^[^ ^176^ ^]^ However, the mechanism of ionic migration in HDPs still needs in‐depth study.

## 5 Recent Developments of Light‐Emitting Diodes Applications Based on Halide Double Perovskites

The colloidal lead halide perovskite nanocrystals with high radiative recombination are well suitable as next generation light emitters, which are favorable to the construction of the high *EQE* and color rendering index (*CRI*) devices.^[^ ^179^, ^180^, ^181^ ^]^ With the consideration of environmental friendliness, lead‐free HDP materials with superior stability urgently need to be developed for LEDs devices applications. The emission from most HDP materials originates from the radiative recombination of STEs. Thus, the HDP materials are suitable to be applied in WLEDs due to its broad emission (**Table** **5**). Manna et al.^[^ ^58^ ^]^ reported that 30% Bi^3+^ doped Cs_2_AgInCl_6_ nanocrystals exhibited double emission at 400–700 nm, which could be used as a single emitter material for WLEDs. The devices were constructed by dispersing nanocrystals in a poly methyl methylacrylate (PMMA) matrix and then multiple coated on glass substrate, which exhibited white light emission with CIE coordinates of (0.36, 0.35), *CRI* values of ≈91 and correlated color temperature (*CCT*) of 4443 K under 380 nm monochromatic UV light. Unfortunately, the researches of colloidal HDP nanocrystals are in the early stage, resulting in little amounts of reports about application in LEDs devices.

**Table 5:** CIE coordinates, *CCT*, and *CRI* of HDP based WLEDs

Device structure	CIE coordinates	*CCT* [K]	*CRI*	Ref.
UV LED/Cs_2_AgIn_0.7_Bi_0.3_Cl_6_ NCs/PMMA	(0.36, 0.35)	4443	≈91	^[^ ^58^ ^]^
UV LED/Cs_2_(Na, Ag)InCl_6_:7.09%Ho^3+^	(0.40, 0.47)	–	75.4	^[^ ^75^ ^]^
UV LED/Cs_2_Na_0.4_Ag_0.6_In_0.995_Bi_0.005_Cl_6_:Mn^2+^	(0.38, 0.42)	4323.4	82.6	^[^ ^77^ ^]^
UV LED/Cs_2_Ag_0.6_Na_0.4_InCl_6_:Bi^3+^	(0.396, 0.448)	4054	–	^[^ ^74^ ^]^
UV LED/Cs_2_AgIn_0.833_Bi_0.167_Cl_6_	(0.448, 0.444)	3119	85	^[^ ^182^ ^]^
UV LED/Cs_2_SnCl_6_:Bi^3+^/Ba_2_Sr_2_SiO_4_:Eu^2+^/GaAlSiN_3_:Eu^2+^	(0.36, 0.37)	4486	–	^[^ ^65^ ^]^
UV LED/Cs_2_AgIn_0.9_Cr_0.1_Cl_6_/SrSi_2_O_2_N_2_:Eu^2+^/CaAlSiN_3_:Eu^2+^	(0.3819, 0.4196)	4200	–	^[^ ^73^ ^]^
NUV LED/Cs_2_ZrCl_6_:Bi^3+^ with silicon coating/Y_3_(Ga, Al)_5_O_12_:Ce^3+^/CaAlSiN_3_:Eu^2+^	(0.37, 0.35)	4179	81.9	^[^ ^68^ ^]^
UV LED/Cs_2_NaInCl_6_:Sb^3+^/Sr_2_Si_5_N_8_:Eu^2+^/*β*‐SiAlON:Eu^2+^	(0.3890, 0.4009)	3972.6	90.6	^[^ ^183^ ^]^
UV LED/Cs_2_Ag_0.4_Na_0.6_InCl_6_:1%Bi^3+^‐1% Ce^3+^	(0.47, 0.45)	2769	84.1	^[^ ^76^ ^]^
UV LED/Cs_2_Ag_0.4_Na_0.6_InCl_6_:1%Bi^3+^‐1% Ce^3+^/BaMgAl_10_O_17_:Eu^2+^	(0.36, 0.33)	4430	95.7	^[^ ^76^ ^]^

The HDP polycrystalline or single crystals with high *PLQY* were widely applied in WLED devices. Generally, combining HDPs with UV LED chips can fabricate WLED devices with high efficiency and *CRI* to produce white light, which mainly involves the preparation of high quality of single white light emitting phosphor. Arfin et al.^[^ ^157^ ^]^ successfully fabricated WLED devices by Cs_2_AgInCl_6_:Bi^3+^‐Er^3+^ phosphors and PMMA on the UV LEDs, resulting in the bright white light emission. However, they did not report the performance of the device. Ho^3+^ doped Cs_2_(Ag, Na)InCl_6_ HDPs with bright warm‐white emission at 490, 450, and 650 nm were reported by Li et al., which had high *PLQY* of 57.09% with Ho^3+^ doping content of 7.09%.^[^ ^75^ ^]^ WLEDs were fabricated by as‐prepared Ho^3+^ doped Cs_2_(Ag, Na)InCl_6_ crystals and 365 nm UV LED chips, which showed the color coordinates of (0.40, 0.47) and *CRI* of 75.4. To obtain low *CCT* and high *CRI* of devices, the Cs_2_Na_0.4_Ag_0.6_In_0.95_Bi_0.05_Cl_6_:Mn^2+^ phosphor with two emission peaks at 550 and 610 nm was used as emitter, which combined with 310 nm UV LED chip to fabricate WLEDs.^[^ ^77^ ^]^ The warm white light could be obtained with CIE coordinates (0.38, 0.42), *CCT* of 4323.4 K and *CRI* of 82.6, indicating that the properties of WLEDs were significantly improved. In addition, Luo et al.^[^ ^74^ ^]^ reported that the Bi^3+^ doped Cs_2_Ag_0.6_Na_0.4_InCl_6_ phosphors with *PLQY* of 86% and UV LED chip (380–410 nm) were used to fabricate WLEDs. They found that the devices with CIE coordinates of (0.396, 0.448) and *CCT* of 4054 K had excellent operational stability under 5000 cd m^−2^ for over 1000 h in air. Gray et al.^[^ ^182^ ^]^ also fabricated a warm WLED with *CCT* of 3119 K and *CRI* of 85 by Cs_2_AgIn_0.833_Bi_0.167_Cl_6_ phosphors with *PLQY* of 39% and UV LED chip. The *CCT* and *CRI* of WLED devices can be significantly improved via using high quality white emission of phosphors.

**Figure 10:**
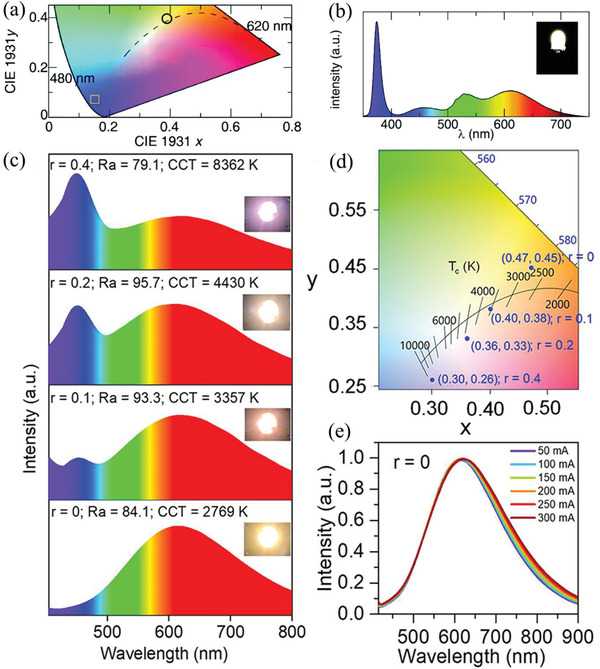
a) CIE coordinates (0.3890, 0.4009) and b) PL spectrum of Cs_2_NaInCl_6_:Sb^3+^ based WLED. The inset is the photo of operating WLED under a forward bias of 20 mA. Reproduced with permission.^[^ ^183^ ^]^ Copyright 2020, The Royal Society of Chemistry. c) Electroluminescence spectra and operating photo of WLED fabricated by different *r* (*r* is the mass ratio of BaMgAl_10_O_17_:Eu^2+^ to 1% Bi^3+^‐1% Ce^3+^ doped Cs_2_Ag_0.4_Na_0.6_InCl_6_). d) CIE coordinates of WLED with different r. e) Electroluminescence spectra of WLED (r = 0) with different driven currents. Reproduced with permission.^[^ ^76^ ^]^ Copyright 2020, American Chemical Society.

On the other hand, the WLEDs fabricated by mixing red, green/yellow, and blue emissions phosphors on the near UV (NUV) or UV LED chips to produce the white light. Tan et al.^[^ ^65^ ^]^ constructed WLED devices by combining Bi^3+^ doped Cs_2_SnCl_6_ vacancy‐ordered double perovskites with the commercial yellow‐emitting phosphors Ba_2_Sr_2_SiO_4_:Eu^2+^ and CaAlSiN_3_:Eu^2+^ as well as a 365 nm LED chip, which showed CIE coordinates of (0.36, 0.37) and *CCT* of 4486 K. In addition, Zhao et al.^[^ ^73^ ^]^ also reported Cr^3+^ doped Cs_2_AgInCl_6_ with the emission peak at 1010 nm due to the broad spin‐allowed ^4^T_2_ → ^4^A_2_ transition of Cr^3+^ ions. The WLED devices fabricated by using Cr^3+^ doped Cs_2_AgInCl_6_ phosphor, green SrSi_2_O_2_N_2_:Eu^2+^ phosphor, and red CaAlSiN_3_:Eu^2+^ phosphor on the 405 nm UV LED chip, which could obtain a WLED with color coordinates of (0.3819, 0.4196) and *CCT* of 4200 K. However, the efficiency of these WLEDs is unsatisfactory. Xiong et al.^[^ ^68^ ^]^ prepared WLED devices via mixing the silane coupling agent encapsulated Bi^3+^ doped Cs_2_ZrCl_6_ HDP materials, green Y_3_(Ga, Al)_5_O_12_:Ce^3+^and red CaAlSiN_3_:Eu^2+^ commercial phosphors on the top of a NUV LED chip. The device exhibited CIE color coordinates of (0.37, 0.35), and CRI of 81.9 and *CCT* of 4179 K. In addition, Gray and co‐workers^[^ ^183^ ^]^ employed blue Cs_2_NaInCl_6_:Sb^3+^ phosphors with emission at 452 nm and *PLQY* of 79%, together with green *β*‐SiAlON:Eu^2+^ phosphors and red Sr_2_Si_5_N_8_:Eu^2+^ phosphors on 370 nm UV LED chip, which exhibited CIE color coordinates of (0.3890, 0.4009), *CCT* of 3972.6 K and *CRI* of 90.6, making Cs_2_NaInCl_6_:Sb^3+^ phosphors as an alternative for commercial blue phosphors (**Figure** **10**a,b). Moreover, Wang and co‐workers^[^ ^76^ ^]^ fabricated WLED devices by Bi^3+^‐Ce^3+^ co‐doped Cs_2_Ag_0.4_Na_0.6_InCl_6_ phosphors with high *PLQY* as well as blue BaMgAl_10_O_17_:Eu^2+^ phosphor. The mass ratio between blue BaMgAl_10_O_17_:Eu^2+^ phosphor and Bi^3+^‐Ce^3+^ co‐doped Cs_2_Ag_0.4_Na_0.6_InCl_6_ phosphors could tune *CCT* from 8362 to 2796 K (Figure 10c). When the mass ratio was 0.2, the devices had high *CRI* of 95.7 and CIE coordinate of (0.36, 0.33) as well as *CCT* of 4430 K (Figure 10d). Furthermore, the WLEDs based on single Bi^3+^‐Ce^3+^ co‐doped Cs_2_Ag_0.4_Na_0.6_InCl_6_ phosphors with a 365 nm UV‐LED chip showed *CRI* of 84.1, *CCT* of 2769 K, and a luminous efficacy of 22.33 lm·W^−1^ at *U* = 3 V and *I* = 100 mA (Figure 10e), which indicated that the high quality HDPs was conducive to obtaining WLED devices with excellent performances.

Although the HDP materials have been demonstrated for application in LEDs owing to its excellent stability and environmental friendliness, there are still some challenges about the applications of HDPs as emissive layers for LEDs with high efficiency and stability. The challenges and resolvable routes are followed: 1) The WLED devices fabricated by using HDPs as emitters still exhibit low luminous efficiency due to a weak absorption and low energy transfer efficiency in blue region.^[^ ^184^, ^185^, ^186^, ^187^ ^]^ Hence, designing highly efficient lead‐free single‐phase full‐color‐emitting HDPs with strong absorption in blue region may boost luminous efficiency of WLED devices. 2) The HDPs emitter with red emission is rarely reported. Hence, developing HDPs with red emission is an important orientation, which can further decrease *CCT* and enhance *CRI* of devices. 3) The emission peak of lead halide perovskite nanocrystals has narrow FWHM, which can be widely applied in high‐resolution and high‐saturation color display. Unfortunately, such a kind of candidate lead‐free HDPs are not reported yet. Hence, it is necessary to discover novel halide perovskite nanocrystals with narrow‐band emission as emitters for meeting the increasing demands on wide‐color‐gamut displays.

## 6 Conclusions and Prospects

The lead‐free HDP nanocrystals offer unique properties, such as nontoxicity, robust intrinsic thermodynamic stability, rich and tunable optoelectronic properties, rendering their promising applications in lighting and display fields. This review comprehensive summarized the background of HDP nanocrystals, and introduced the crystalline structure, electronic structure, and PL mechanism, followed by analyzing the limiting factors on optical properties and the sources of instability and discussed the effects of synthesis strategies, ligands passivation and metal doping/alloying on the PL properties, and stability of the HDPs. Finally, we outlined their preliminary applications for LED devices. Particularly, synthesis, ligand, and metal doping/alloying strategies in HDP nanocrystals have been summarized as follows: First, for synthesis strategies, the hot injection technique has been widely applied to prepare various nearly monodisperse HDP nanocrystals with high crystallinity, including Cs_2_AgBiX_6_ (X = Cl, Br),^[^ ^51^ ^]^ Cs_2_AgSbX_6_ (X = Cl, Br),^[^ ^121^, ^128^ ^]^ Cs_2_NaBiX_6_ (X = Cl, Br),^[^ ^124^, ^125^, ^126^ ^]^ Cs_2_AgInCl_6_,^[^ ^127^, ^128^ ^]^ and Cs_2_NaInCl_6_^[^ ^130^ ^]^ nanocrystals. At the same time, this route can effectively adjust the shape by precisely controlling reaction condition, which is suitable to produce and explore new‐type lead‐free HDP nanocrystals, such as Cs_2_CuSbCl_6_ nanocrystals.^[^ ^131^ ^]^ However, hot injection method need to be performed in an air‐free atmosphere for air sensitive precursors and the resultant products exhibit low *PLQY* owing to surface defects. These problems can be overcome by performing antisolvent recrystallization method, which can easily be used to produce HDP nanocrystals with high *PLQY* under air atmosphere. Unfortunately, antisolvent recrystallization route is based on metal halide salt and polar solvents, which might degrade the as‐prepared lead‐free HDP nanocrystals, resulting in the decline of stability. Second, for ligand strategies, the OA and OLA have been widely used as ligands to prepare HDP nanocrystals. Commonly, OLA acted as a complexing agent for metal ions, while OA played a role in suppressing crystal growth during the synthesis of HDP nanocrystals. However, large amounts of OLA easily reduce Ag^+^ to Ag^0^, leading to poor stability of Cs_2_AgInCl_6_ nanocrystals.^[^ ^122^ ^]^ Similarly, OLA can reduce Cu^2+^ to Cu^+^ as precursor for prepared Cs_2_CuSbCl_6_ nanocrystals because the OLA can provide a weak reduction condition.^[^ ^131^ ^]^ In addition, alone OA as ligand can effectively passivate surface of HDP nanocrystals, thereby resulting in boosting stability and PL intensity.^[^ ^137^ ^]^ Finally, for metal doping/alloying strategies, the different valence states of B‐site metals in HDP structure provide more possibility of doping/alloying from monovalent to quadrivalent metal ions, generating fascinating photoelectricity properties in the target materials. For example, the monovalent or trivalence metal cation doping in A_2_B^+^B^3+^X_6_ can tune bandgap and electronic structure. The lanthanide ions and Mn^2+^ ions doped HDP structure are both demonstrated multi‐peak PL emission from the energy transfer between host energy levels and guest energy levels. These reported strategies have been shown to explore and prepare highly efficient HDP nanocrystals, modulate their PL properties, and boost their stability. However, the *PLQY* and PL intensity of the HDP nanocrystals remained dissatisfactory compared with lead‐based halide perovskites nanocrystals. We envisage that the following aspects will be important for obtaining lead‐free HDP nanocrystals and fabricating LED devices with desirable properties in future research.

i) Developing effective and versatile synthesis strategies for HDP nanocrystals. Owing to the easy oxidization of Cu^+^ and In^+^, many lead‐free HDP nanocrystals (such as Cs_2_CuSbCl_6_, Cs_2_InBiCl_6_) with fascinating performance by discovering theoretically are difficult to prepare. Moreover, the lead‐free perovskite nanocrystals with narrow‐broad emission have hardly been reported. Hence, the effective and versatile synthesis strategies are deserved development to prepare high quality broad emission of HDP nanocrystals toward solid‐state lighting application and narrow‐band emission of lead‐free perovskite nanocrystals for display application. In addition, deepening the understanding of the formation mechanism for colloidal HDP nanocrystals can significantly design and guide for the synthesis of high‐quality nanocrystals. Furthermore, different shapes of colloidal HDP nanocrystals have important influence on optoelectronic properties and stability, which have been demonstrated in lead halide perovskite nanocrystals,^[^ ^187^, ^188^ ^]^ thus, the shapes further need to be controlled precisely by adjusting reaction conditions.
ii) In‐depth understanding of ligand mechanism for colloidal HDP nanocrystals. At present, knowledge about ligand chemistry of lead‐free HDP nanocrystals is insufficient. For instance, the passivation mechanism of capping ligands has not been investigated in depth for boosting efficiency and stability of lead‐free HDP nanocrystals. For Bi^3+^ doped Cs_2_Ag_1−_ *_x_*Na*_x_*InCl_6_ nanocrystals, the insufficient Cl^−^ ions on nanocrystals surface would lead to deep trap states, which were different from lead halide perovskite with highly defect tolerance.^[^ ^189^ ^]^ In addition, to avoid the addition of oleylamine, the ligands with strong anchoring groups can be used to passivate surface of HDP nanocrystals for improving stability and *PLQY*, which is conducive to facilitate tight binding between ligands and nanocrystals. The functional ligands also have a great probability to further modify properties of HDP nanocrystals by judicious molecular design, which will be beneficial for further functionalization of the lead‐free HDP nanocrystals.
iii) Tailoring the HDP nanocrystals compositions precisely via metal doping/alloying strategies. Current research on doping/alloying HDP nanocrystals is still in the infancy stages. Composition engineering via more efficient dopants or co‐doping strategies in different sites will become a significant research direction, which can boost or modulate the efficiency and stability of HDP nanocrystals for the wishful devices. In addition, the interrelations of electronic structure and PL properties between doped ions and HDP hosts need to be explored.
iv) Developing efficient and stable lead‐free HDP nanocrystals for WLEDs devices. The lead‐free HDP nanocrystals are very suitable for the application in WLEDs due to their broadband visible emission. However, rarely literature reported that HDP nanocrystals were used to fabricate WLEDs. In addition, although many HDP materials have been successfully applied in WLEDs with superior operational stability, lead‐free HDP materials as the emitting materials still faces great challenges. Thus, it makes sense to design highly *PLQY* and stable lead‐free HDP nanocrystals and fabricate WLED devices with high luminous efficiency, CIR, long lifetime, and stability. For example, developing HDP nanocrystals emitter with red emission can obtain devices with low *CCT* and high *CRI*. In addition, the colloidal HDP nanocrystals possess excellent low‐temperature solution processability. Hence, the electroluminescence devices via HDP nanocrystals as the emitting active layers are important developmental direction for the solid‐state lightings and displays application.
v) Enhancing stability of HDP nanocrystals. The stability remains mainly challenging, either for HDP nanocrystals or for lead halide perovskite nanocrystals, which includes colloidal stability, phase stability, light stability, humidity stability, oxygen stability, and thermal stability. The chloride double perovskite nanocrystals with excellent stability have been demonstrated via previously reports, making them possess outstanding operational stability in LEDs devices. Nonetheless, bromide/iodide‐based double perovskite nanocrystals or HDP nanocrystals with both lone‐pair states in B^+^/B^3+^ sites still suffer degeneration issues due to its low thermodynamic stability. The stability of HDP nanocrystals can be boosted by the following strategies: 1) The construction core‐shell structures or surface modification via organic or inorganic matrix to prevent nanocrystals from directly contacting with the external environment. 2) Adjusting composition to change the tolerance factor, decrease intrinsic defects density, and increase ionic size matches.

In summary, tremendous progress has been made in improving properties of colloidal HDP nanocrystals, which exhibits fascinating efficiency and stability for application in LEDs. The synthesis strategies, ligands passivation, and metal doping/alloying can efficiently boost performance of colloidal halide HDP nanocrystals. However, the fabrication of highly efficient and stable LEDs devices often requires the synergistic effect of several strategies. With sustained efforts in designing high‐quality colloidal nanocrystals and modulating their properties to obtain desired functionality, lead‐free HDP nanocrystals offer a promising route to manufacture high efficiency and stability of LEDs in future.

## Conflict of Interest

The authors declare no conflict of interest.
